# Polyethylene glycol-based deep eutectic solvents as a novel agent for natural gas sweetening

**DOI:** 10.1371/journal.pone.0239493

**Published:** 2020-09-21

**Authors:** Jiyad N. Aldawsari, Idowu A. Adeyemi, Abdelbasset Bessadok-Jemai, Emad Ali, Inas M. AlNashef, Mohamed K. Hadj-Kali

**Affiliations:** 1 Department of Chemical Engineering, King Saud University, Riyadh, Saudi Arabia; 2 King Abdulaziz City for Science and Technology, Riyadh, Saudi Arabia; 3 Department of Chemical Engineering, Khalifa University, SAN Campus, Abu Dhabi, United Arab Emirates; University of South Carolina, UNITED STATES

## Abstract

Deep eutectic solvents (DESs) have received significant attention as potential extracting agents in recent years due to their favorable characteristics including low cost, easy preparation and environmentally safe starting materials. Experimentally screening for highly efficient DESs meeting various requirements for natural gas sweetening remains a challenging task. Thus, an extensive database of estimated Henry’s law constants (*H*_*i*_) and solubilities (*x*_*i*_) of CO_2_ in 170 different DESs at 25°C has been constructed using the COSMO-RS method to select potential DESs. Based on the COSMO-RS study, three DESs, namely tetrabutylammonium bromide (TBAB)+polyethylene glycol (PEG-8) (on a molar basis 1:4), TBAB+octanoic acid (OCT) (1:4), and methyltriphenylphosphonium bromide (MTPB)+PEG-8 (1:10), were chosen for further experimentation up to 2 bar at 25°C using a vapor-liquid equilibria (VLE) apparatus. Reliable thermophysical properties were determined experimentally, and a detailed equilibrium-based model was developed for one of the glycol-based DESs (i.e., TBAB+PEG-8 (1:4)). This information is an essential prerequisite for carrying out process simulations of natural gas sweetening plants using ASPEN PLUS. The simulation results for the proposed DES were compared to those of monoethylene glycol (MEG). Here, we find that the aqueous TBAB+PEG-8 (1:4) solvent shows ~60% lower total energy consumption and higher CO_2_ removal when compared to those using the MEG solvent.

## Introduction

The global climate has witnessed severe changes in the last decade primarily due to greenhouse gases released by the combustion of coal, oil, and natural gas (NG) [1]. Among the greenhouse gases, CO_2_ is major contributor to the global warming, with the lion’s share of 42%, equivalent to 14.2 Gigatons (Gt), emitted from the power sector alone [2, 3]. Furthermore, the energy-related CO_2_ emissions are expected to increase during the next decade according to the World Energy Outlook (WEO) [4]. This outlook also predicts an accelerated increase in the consumption of NG as an alternative, less-polluting fuel based on emission requirements established by environmental regulatory agencies [4, 5]. Thus, there is an urgent need for technologies that can better utilize NG and improve the process to meet regulatory standards.

Various CO_2_ capture technologies have been proposed since the 1930s, including physical absorption, chemical absorption, and most recently membranes [6, 7]. Chemical absorbents, especially amine-based solvents, are the most-used method for removing CO_2_ from NG in a process known as “gas sweetening” due to their low cost and high CO_2_ loading. However, the large enthalpy of reaction, corrosivity, and volatility increase the capital cost (CAPEX) and operating cost (OPEX) of the entire capture system [3, 8]. For these reasons, there is a quest for a capture process with less cost and energy requirements.

Physical absorption using physical solvents (e.g., Selexol^®^, Morphysorb^®^, Rectisol^®^, Purisol^®^, and most recently ionic liquids) is a viable alternative to chemical absorption. The main advantage of physical solvents over aqueous alkanolamines solutions is the lower energy requirement as CO_2_ absorption is accomplished through physical interactions without chemical reactions [9, 10]. However, this type of technology has several drawbacks as well, including low solubility and selectivity toward CO_2_ [9]. Ionic liquids (ILs), however, can be tailored through the appropriate selection of cations and/or anions to obtain any desirable properties (e.g., high selectivity and solubility), which makes them a solvent of interest [3, 11]. For large-scale applications, drawbacks of ILs include high cost, unidentified toxicity, poor biodegradability, and complicated production and purification technologies in addition to their high viscosity [12, 13].

**Fig 1:**
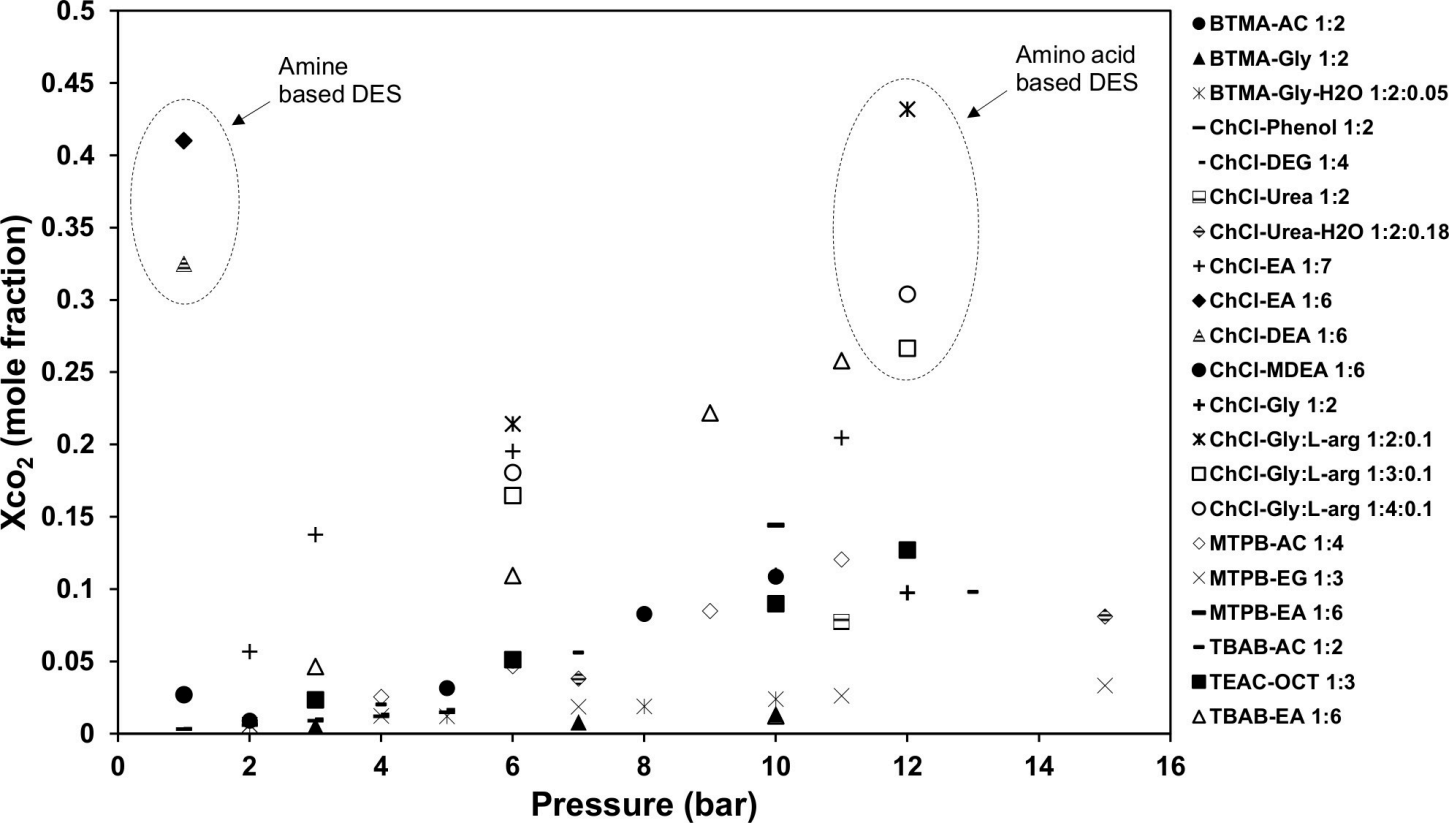
CO_2_ solubility in various DESs reported at temperature range from 298.15–313.15K. (**●**) Benzyl trimethylammonium chloride (BTMAC)+acetic acid (AC) (1:2) [11], (**▲**) BTMAC+glycerol (Gly) (1:2) [11], (Җ) BTMAC+Gly+H_2_O (1:2:0.05) [11], (**−**) Choline chloride (ChCl)+phenol (1:2) [19], (**-**) ChCl+diethylene glycol (DEG) (1:4) [11], (□) ChCl+urea (1:2) [15], (◊) ChCl+urea+H_2_O (1:2:0.18) [17], (+) ChCl+ethanolamine (EA) (1:7) [11], (**♦**) ChCl+EA (1:6) [21], (Δ) ChCl+diethanolamine (DEA) (1:6) [21], (**○**) ChCl+ Methyldiethanolamine (MDEA) (1:6) [21], (+) ChCl+Gly (1:2) [20], (**Җ**) ChCl+Gly+L-arginine (1:2:0.1) [22], (**□**) ChCl+Gly+L-arginine (1:3:0.1) [22], (**○**) ChCl+Gly+L-arginine (1:4:0.1) [22], (◊) Methyltriphenylphosphonium bromide (MTPB)+AC (1:4) [11], (X) MTPB+ethylene glycol (EG) (1:3) [11], (**−**) MTPB+EA (1:6) [11], (**-**) Tetrabutylammonium bromide (TBAB)+AC (1:2) [11], (**■**) Tetraethylammonium chloride (TEAC)+OCT (1:3) [11], (**Δ**) TBAB+EA (1:6) [11].

Deep eutectic solvents (DESs) are promising sustainable alternatives to ILs. They share the same advantageous features of ILs including negligible vapor pressure, non-flammability, and high thermal stability; in addition to their lower price compared to ILs, biodegradability, and simple preparation [11, 14]. The solubility of CO_2_-DESs systems has been investigated in many studies recently. For example, choline chloride-based DESs have been widely investigated and reported in the literature as potential CO_2_ capture media. Li and co-workers [15] determined the solubility of CO_2_ in a choline chloride (ChCl) + urea DES at different molar ratios, temperatures, and pressures. Others used the same mixture at a moderate CO_2_ pressure of 10 bar to investigate the effect of water on the solubility of the mixture [16, 17]. The results from these investigations reveal low CO_2_ capture ability, i.e., up to 0.3–5 mol% (Fig 1) under near-ambient operating conditions, which is clearly unsatisfactory for industrial purposes, especially for flue gas treatment [18].

The influence of hydrogen bond donors (HBDs) on the absorption of CO_2_ was also investigated as amine- and amino acid-based HBDs seem to perform better than other HBDs. One example is for a DES consisting of ChCl+EA at a 1:6 molar ratio (Fig 1), whereby Adeyemi *et al*. [21] have reported high absorption capacity at ambient pressure, while the highest value is 0.432 mol CO_2_/mol DES reported for ChCl-Gly: L-arg (1:2:0.1) DES at ~ 12 bar. This performance was validated by the formation of hydrogen bonds between O–H and N–H of the individual DES components, and the chemical absorption due to the inclusion of the amine. A second example is for ternary DESs consisting of a mixture of ChCl + glycerol + L-arginine, as reported by Fareeda *et al*. [22]. The addition of 0.1 mole of L-arginine to the glycerol-based DES enhanced the solubility of CO_2_ by 300%. However, the viscosity of this DES increased sharply, and a noticeable reduction in solubility was observed when the quantity of L-arginine was increased further.

Thus, precise selection of the DES building blocks is an essential task for developing the optimal CO_2_ capture process. However, this is a non-trivial task due to the essentially limitless number of possible choices. Moreover, it is unreasonable to conduct time-consuming and expensive experiments just to confirm the suitability of a certain class of DESs. Consequently, there has been significant growth in the application of predictive models for estimating thermodynamic properties. Such methods are crucial for the design of new DESs with specific industrial applications, expanding the rapidly growing set of available or potentially available DESs.

There are many types of computational methods to calculate and predict the properties of DESs, namely, quantitative structure–property relationships (QSPR), equations of state, artificial intelligence (AI) algorithms, molecular simulations (MS), the quantum chemistry-based conductor-like screening model for real solvents (COSMO-RS) method, and other classical thermodynamic models, such as NRTL, UNIQUAC, and UNIFAC [23]. In terms of thermodynamic calculations, the COSMO-RS model has been reported to produce good qualitative results and satisfying quantitative predictions of activity coefficients of neutral compounds in ILs [24]. The same model was also used by Hanee [25] to screen potential DESs for the removal of aromatic nitrogen compounds from diesel fuel. The results suggested the practical use of COSMO-RS as a preliminary screening tool rather than for design calculations. Lei *et al*. [26] investigated the applicability of a macroscopic thermodynamic model to predict the solubility of CO_2_ in ionic liquids. They verified that the UNIFAC model is suitable for predicting CO_2_ solubility at either high or low temperatures. Ali *et al*. [20] applied the Peng–Robinson (PR) equation of state to investigate CO_2_ solubility in synthetic phosphonium- and ammonium-based DESs. The results show good agreement between this model and the experimentally reported data. Zubeir *et al*. [27] applied perturbed-chain statistical associating fluid theory (PC-SAFT) to describe the phase behavior of DES + CO_2_ systems in the temperature range of 298.15 and 318.5 K and pressures up to 20 bar. The PC-SAFT model was in all cases able to model the VLE data correctly. Xie *et al*. [28] examined the absorption of CO_2_, CH_4_, CO, and N_2_ in ChCl+urea, where non-random two-liquid and Redliche Kwong (NRTL-RK) thermodynamic models were used to fit the solubility of CO_2_ in ChCl+urea.

The objective of the present study is to investigate the potential use of DESs as physical solvents for CO_2_ capture. Thus, a predictive modeling technique based on the quantum chemistry method is proposed for screening 170 novel DES combinations in order to select a new class of DESs with high performance. Furthermore, experiments were conducted to validate the VLE data and to identify certain thermophysical parameters (e.g., density, viscosity) needed to establish a successful process of interest. Moreover, conceptual process layouts for capturing CO_2_ from crude NG were simulated using the ASPEN PLUS simulator (V10.1) from Aspen Technology, Inc. (Cambridge, MA). The simulation results were validated by comparison with benchmark physical solvents (i.e., mono ethylene glycol, MEG).

## Materials and method

### Materials

Chemicals used in this work involve the salts: tetrabutylammonium bromide (C_16_H_36_BrN); methyltriphenylphosphium bromide (C_19_H_18_BrP); as well as several glycol based HBDs. All chemicals were utilized without further purification. Details of the list of chemicals used, their purity and abbreviation are presented in Table 1.

**Table 1:** CAS registry number and mass fraction purity of the chemicals used in this work.

Chemical	Abbr.	CAS No.	Purity	Supplier
Tetrabutylammonium bromide	[TBAB]	1643-19-2	>0.998	Loba Chemie, India
Methyl triphenyl phosphonium bromide	[MTPB]	1779-49-3	>0.988	Acros Organics, India
Octanoic acid	[OCT]	124-07-7	>0.999	Sigma Aldrich, USA
Poly-ethylene glycol	[PEG-8]	25322-68-3	>0.999	Sigma Aldrich, USA

### Synthesis of the DESs

The DESs were prepared by direct mixing of salts and HBDs according to the specified molar ratio. Each salt was mixed with the corresponding HBD using magnetic stirring at 400 rpm and 353.15 K until a homogenous transparent liquid is formed. Afterward, the mixture was placed in a moisture-controlled area to cool down at room temperature. The weight of the materials was determined by analytical balance (Mettler Toledo AL204) with the standard uncertainty of 10^−4^ g. The water content of all DESs was determined by Karl-Fischer titration analysis (Aquamax Karl-Fischer titration, GR Scientific Ltd.), resulting in mass fractions of < 1200 ± 100 ppm for all DESs. The DESs prepared with different salt to HBD molar rations are summarized in Table 2.

**Table 2:** Molar ratios, abbreviations, appearance and melting points of the TBAB-base DES.

DESs Molar ratio	Abbr.	Appearance at room temperature	Melting points (K)^a^
1 TBAB: 4 PEG-8	TBAB+PEG-8 (1:4)	Colorless liquid	267.24
1 TBAB: 4 OCT	TBAB+OCT (1:4)	Colorless liquid	232.67
1 MTPB: 10 PEG-8	MTPB+PEG-8 (1:10)	Colorless liquid	-

^a^ Standard uncertainty u in measured melting temperature is u(T) = 0.01 K.

### CO_2_ absorption experiment

**Fig 2:**
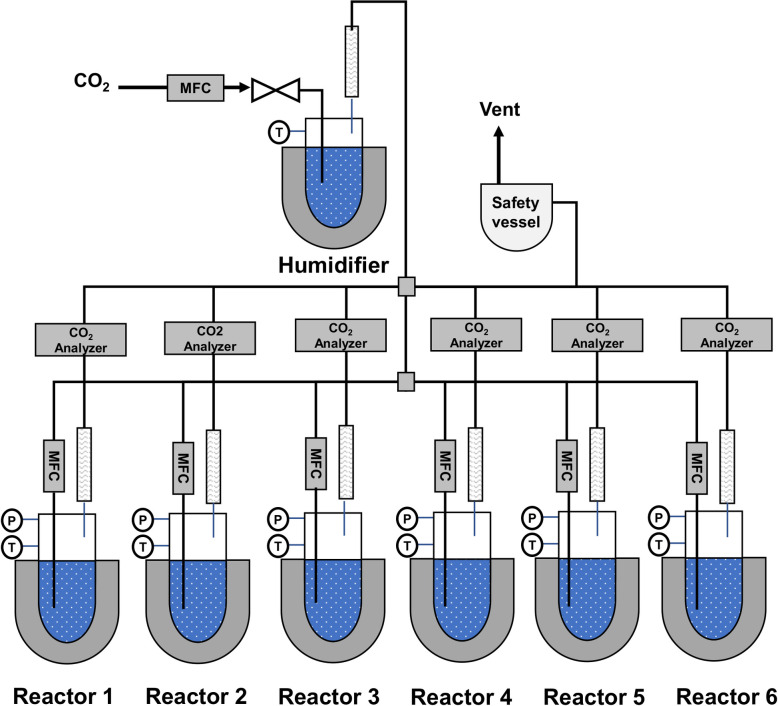
Schematic diagram of CO_2_ absorption apparatus [29]. (MFC stands for mass flow controller).

The experimental measurements of the solubility of CO_2_ in the three DESs with highest solubility was conducted with the Solvent Screening Set-Up (Fig 2). The solvent screening set-up is a bench-scale laboratory equipment which consists of six batch process reactors. The CO_2_ absorption experiment was initiated by filling the reactor vessel with DES to the 200 mL mark, and purging the empty air space with nitrogen in order to eliminate any pre-existing gases in the system before the experiment. Then, CO_2_ was fed into a make-up vessel to a pressure of 2 atm, after all residual gases have been eliminated by the N_2_, to ensure that enough pressure is attained and sustained in the reactors. The CO_2_ absorption was observed at 1.01 bar and 40°C, and the experiment was stopped after the DES was saturated with the CO_2_. Thereafter, the measurement of the CO_2_ in the DESs was achieved with an Elementar Total Organic Content (vario TOC cube) analyzer with high temperature catalytic combustion at 850°C and non-dispersive infra-red detector (NDIR).

## Model development

### COSMO-RS model development

COSMO-RS is a thermodynamic model based on quantum chemistry that can be used to estimate the chemical potential of molecules in liquids; In addition, other properties such as solubility, activity, and vapor pressure can be calculated using chemical potential [30]. The attractiveness of this method is that it is purely predictive, based on first principles, and does not require group parameters or any system-specific adjustments [23, 31]. Detailed explanations on how the COSMO-RS calculations are performed are given in references [24, 25, 29, 31, 32]. COSMO-RS calculations involve two steps: (i) Molecular geometry optimization using density functional theory where the three-dimensional distribution of the screening charge density (*σ)* on the surface of each molecule is obtained by quantum calculation, and (ii) defining chemical potentials based on statistical thermodynamics principles using information from the resulting local polarization charge densities *σ* and probability densities *P*_*i*_*(σ)* [23].

### DES database and computational model details

**Fig 3:**
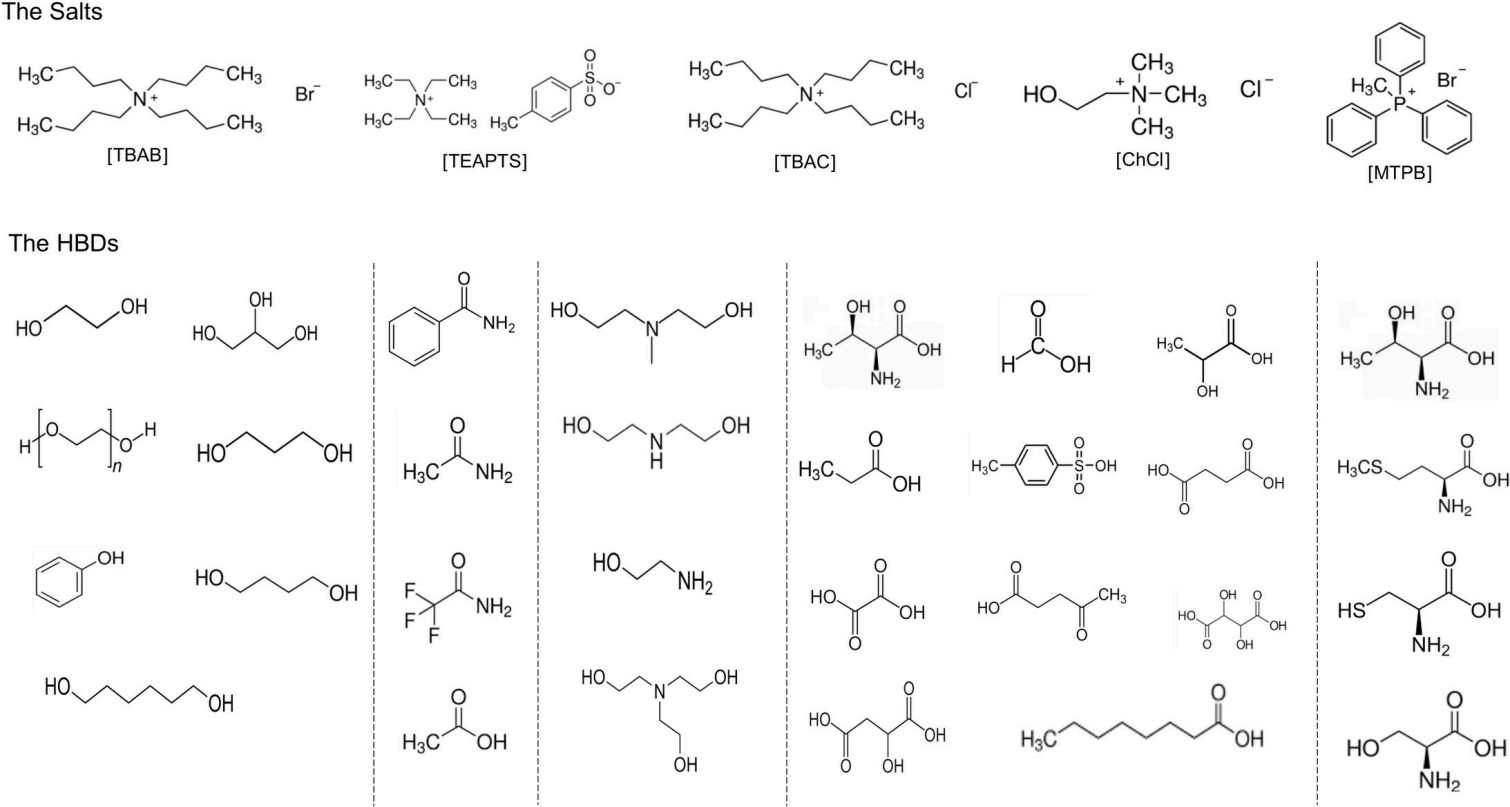
Structures of the salts and HBDs of DESs. (A) alcohols-based HBD, (B) amide-based HBD, (C) amine-based HBD, (D) organic acid-based HBD, (E) organic amino acid HBD. (*n* = 2 (DEG), 3 (TEG), 4 (PEG-4) and 8 (PEG-8)).

Five salts and 34 HBDs (reported in Tables 3 and 4) were investigated in this work. As a result, the molecular geometries of 170 DESs were optimized by density functional theory and the Becke–Perdew functional with triple-zeta valence polarized (BP-TZVP) basis using TURBOMOLE V-3.4 software to generate the cosmo files [33]. TZVP parameterization was chosen because it gives more meaningful values in terms of hydrogen bonding interactions, which is a vital interaction between the CO_2_ compound and DESs [25]. Two groups of salts, quaternary ammonium (NR^4+^) and quaternary phosphonium (PR^4+^), and five different groups of HBDs, alcohols, amines, amides organic acids, and amino acids, were used to construct the DES database (Fig 3). All thermodynamic calculations were performed with COSMO*therm* using the C21-0108 parameterization.

**Table 3:** List of salts used in this work to create the DESs database with abbreviation and molecular weight (*M*_*W*_).

Group	Chemical	Abbr.	*Mw* (g.mol^-1^)
NR^4+^	Tetrabutylammonium bromide	[TBAB]	322.36
	Tetrabutylammonium chloride	[TBAC]	277.92
	Tetraethylammonium *p-*toluene sulfonate	[TEAPTS]	301.44
	Choline chloride	[ChCl]	139.62
PR^4+^	Methyltriphenylphosphonium bromide	[MTPB]	357.22

**Table 4:** List of HBDs used in this work to create the DESs database with abbreviation and molecular weight (*M*_*W*_).

Group	Chemical	Abbr.	*Mw* (g.mol^-1^)	Group	Chemical	Abbr.	*Mw* (g.mol^-1^)
Alcohol	Ethylene glycol	[MEG]	62.07	Organic acids	Levulinic acid	[LV]	116.11
	Diethylene glycol	[DEG]	106.12		*P*-toluene sulfonic acid	[PTSA]	172.2
	Triethylene glycol	[TEG]	150.17		lactic acid	[LA]	90.08
	Tetraethylene glycol	[PEG-4]	194.23		Octanoic acid	[OCT]	144.21
	Polyethylene glycol	[PEG-8]	400.11		Tartaric Acid	[TA]	150.08
	Glycerol	[Gly]	92.09		Formic acid	[FA]	46.03
	Phenol	[Phenol]	94.11		Malic Acid	[MA]	134.08
	1,4-butanediol	[1,4 BDO]	90.12		Oxalic Acid	[OXA]	90.03
	1,6-hexanediol	[1,6 HDO]	118.17		Acetic acid	[AC]	60.05
	1,3-propanediol	[1,3 PDO]	76.09		Succinic acid	[SA]	118.09
Amides	Acetamide	[DMA]	59.07		Propionic acid	[PA]	74.08
	Benzamide	[BA]	121.14		Benzoic acid	[BA]	122.12
	2,2,2Triflouracetamid	[C_2_H_2_F_3_NO]	113.03		Glutamic Acid	[GA]	147.13
	Urea	[Urea]	60.06	Amino acid	L-threonine	[L-Theo]	119.11
Amines	Ethanolamine	[MEA]	61.08		L-serine	[L-Seri]	105.09
	Diethanolamine	[DEA]	105.14		L-cysteine	[L-Cyst]	121.16
	Triethanolamine	[TEA]	149.18		L-methionine	[L-Meth]	149.21
	Methyldiethanolamine	[MDEA]	119.16				

Representative DESs can be obtained by one of the three approaches: (i) the metafile approach, (ii) the ion pair approach, and (iii) the electro-neutral approach [23, 34]. In this study, the third approach, whereby DESs are considered as completely dissociated ions (cation and anion) with respect to their mole ratio, was adopted. Indeed, in liquid form, DESs can be viewed as three distinct species, i.e., salt cation, salt anion, and HBD. Hence, a DES with a salt:HBD molar ratio of 1:*n* is represented by 1 mole of salt cation, 1 mole of salt anion, and *n* mole of HBD. Therefore, the activity coefficients, gas solubilities, and Henry’s law constants obtained directly from COSMO*therm* (*log*_*10*_ *γ*_*i*_, *log*_*10*_ *x*_*i*_, *H*_*i*_) are converted to a binary framework by scaling with their equivalent molar ratio, i.e., 1:1:*n* for the cation:anion:HBD ratio to make them comparable with experimental results where the DES is considered a single entity. The screening was conducted at 25°C and 1 bar. Moreover, COSMO*therm* was used to predict the solubility of light hydrocarbon gases (i.e., CH_4_, C_2_H_6_, and C_3_H_8_) and hydrogen sulfide (H_2_S) with the same DESs to determine those with high selectivity and high capacity for various gases. In fact, the selectivity of a given solvent for one gas (denoted as *a*) to another (denoted as *b*) can be calculated using Eq (1).

$$S_{\left(a/b\right)}=\frac{H_{b}}{H_{a}}$$ (1)

### Aspen plus model development

After COSMO-RS screening and experimental evaluation, the selected DES was examined by simulating the conceptual CO_2_ capture process via ASPEN PULS software. The conceptual capture process is a typical absorption-desorption process. Because DESs are not available in the ASPEN PLUS database, they have been defined as pseudo-components by defining their physicochemical properties. The process simulations were performed in the equation-oriented mode, which solves mass and energy balances while simultaneously avoiding nested convergence loops and is more effective for processes containing recycling streams and design specifications than those of the sequential modular mode [35]. The objective of the simulation was to test the effectiveness of the selected DESs in a closed loop process. This helps in estimating the inventory and energy requirement for a typical industrial-scale CO_2_ capture process.

#### Process description

**Fig 4:**
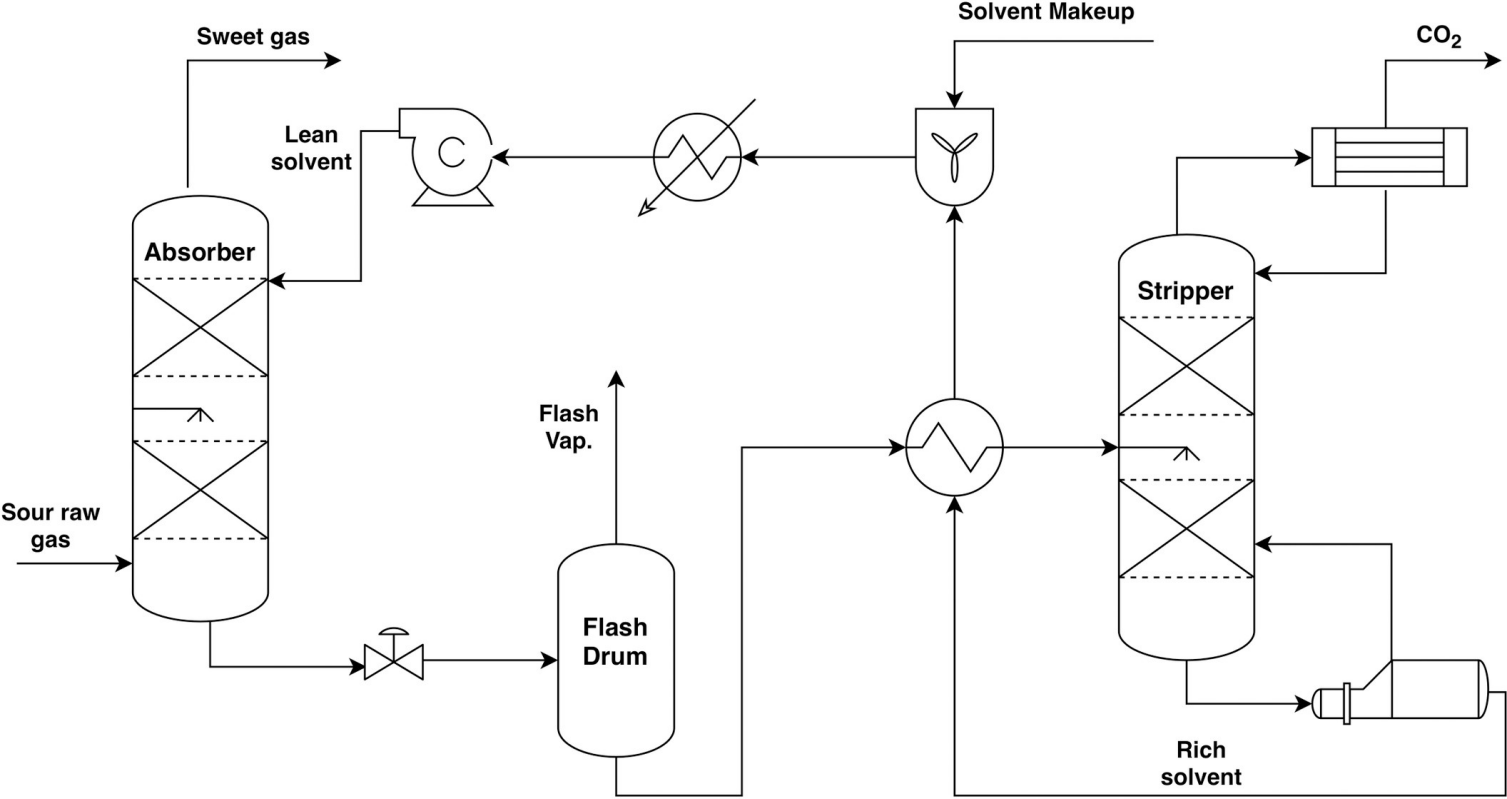
Scheme of the natural gas sweetening process.

A schematic of the process for a gas sweetening unit with MEG in a large-size NG plant is shown in Fig 4.

The gas operating conditions and compositions selected in this study are listed in Table 5. The process mainly consists of absorption and regeneration columns. Sour gas flows into the bottom stage of the absorber at 30°C. The lean MEG solution (80 wt%) absorbs CO_2_ from the gas by forming weakly bonded compounds called carbamate while flowing in a counter-current manner.

**Table 5:** Sour gas conditions (adopted from [36]).

Parameters	Unit	Value
Feed Temperature	°C	30.0
Absorber Pressure	bar	52
Sour gas Flowrate	kmol.h^-1^	5000
Lean solvent rate	m^3.^h^-1^	800
Sour gas Component mole fraction		
Hydrogen sulfide	mol%	2.90
Carbon dioxide	mol%	10.0
Nitrogen	mol%	0.10
Methane	mol%	72.0
Ethane	mol%	7.00
Propane	mol%	5.00
n-Butane	mol%	0.70
i-Butane	mol%	0.70
n-Pentane	mol%	0.60
Water vapor	mol%	1.00

The treated gas exits from the top of the absorber while the CO_2_-rich solvent exits from bottom. The rich solvent leaving the absorber flows to the flash drum to remove any impurities that might cause problems for the equipment in this process. The rich solvent leaves the flash drum to the rich/lean heat exchanger where heat is absorbed from the lean solution. The heated rich solvent flows into the stripper where CO_2_ is regenerated from the solvent through heat input to the reboiler. The lean solvent exits the bottom of the regeneration column and is recycled to the absorber passing through the rich/lean exchanger, cooler, and pump. Makeup solvent is added to the recycled lean solvent as well, which accounts for any loss of solvent.

#### Process simulation

**Fig 5:**
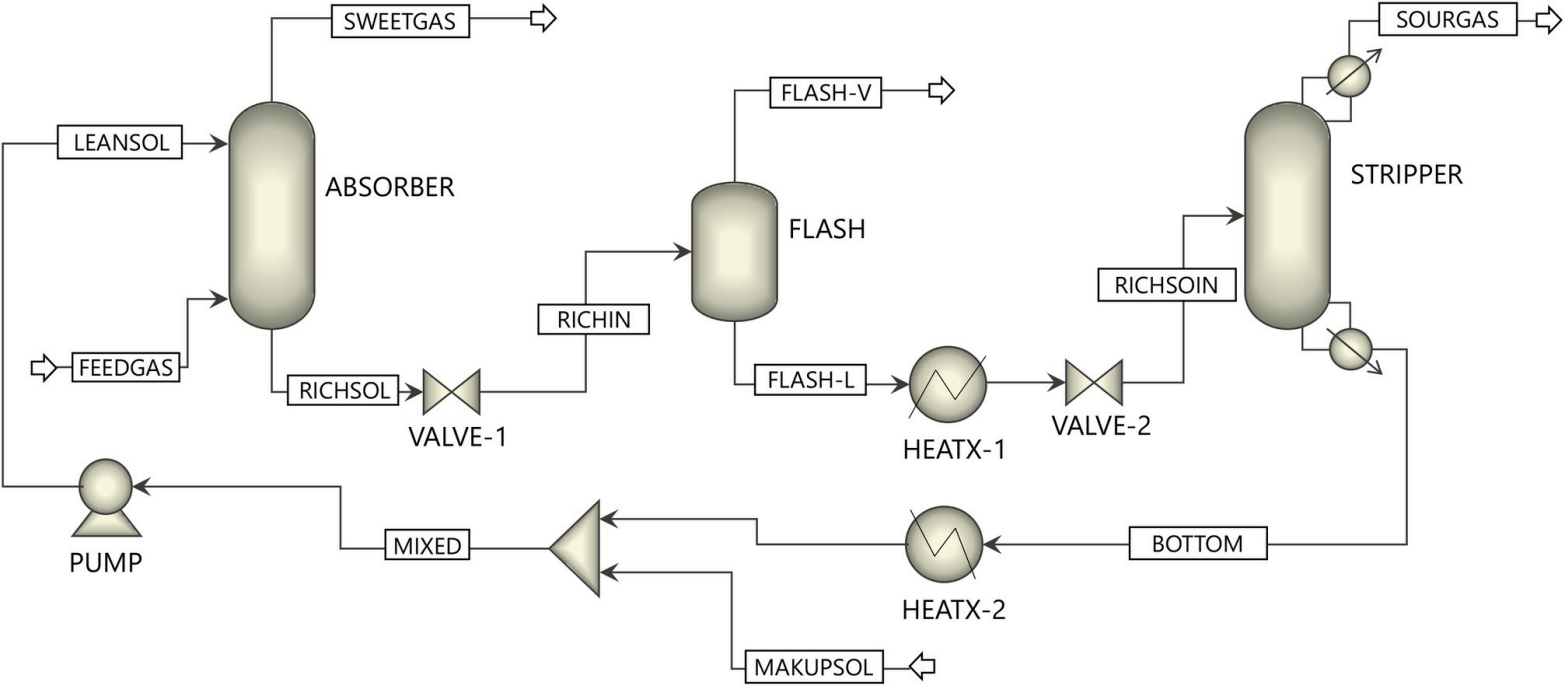
Scheme of the simulated plant (as from ASPEN PLUS).

The CO_2_ capture process is simulated using the latest version of ASPEN PLUS (V10.1), which generates the flow sheet shown in Fig 5.

In this process, the DES is defined as a pseudo-component by specifying its molecular weight, normal boiling temperature, and density (S2, S3 and S4 Tables in S1 File). Additionally, the coefficients of the Andrade’s equation for the viscosity-to-temperature dependency were specified. These coefficients were regressed from experimental data obtained in this work (S5 Table in S1 File). The remainder of the unknown properties including pseudo-critical properties, molar heat capacity, enthalpy of vaporization, and liquid vapor pressure of the DES pseudo-components were estimated by the methods and models implicitly used in Aspen Plus (S1, S6, S7, S8 and S9 Tables in S1 File); the same methodology was developed for IL_S_ [35]. To the best of our knowledge, there are no publication regarding the process simulation of this family of DES.

The absorber and stripper models were developed using the RADFRAC model in ASPEN PLUS. There are two main approaches to modeling absorber and desorber in ASPEN PLUS: (i) equilibrium and (ii) rate-based approaches. In this work, the equilibrium approach was used. In the equilibrium-based model, the liquid and gas phases are assumed to be in equilibrium [37]. The process depicted in Fig 5 consists of two heat exchangers (HEATX-1 and HEATX-2) that collectivity represent the heat exchangers and coolers shown in Fig 4, two valves (VALVE-1 and VALVE-2) to regulate the pressure within the gas sweetening unit, the recycling pump (PUMP), and the flash drum (FLASH) implicitly used in the MEG process to avoid problems caused by impurities and to remove some of the absorbed hydrocarbons [36].

#### Thermodynamic model

The COSMO-segment activity coefficient (COSMO-SAC) thermodynamic model developed by Lin and Sandler in 2002 and then embedded in ASPEN was selected in this work to estimate the activity coefficients of the components in the mixtures [38]. The molecular volumes and *σ*-profiles of the DESs needed for specifying the COSMO-SAC model were obtained from the previous COSMO-RS calculations by the COSMO*therm* (v C21_0108) program package assuming ion-paired structures (i.e., metafile approach). It must be noted that, in ASPEN PLUS, the COSMO-SAC model does not require binary parameters to account for the interaction between components but requires six input parameters that are genuine for the COSMO-SAC model for each component [39]. The component volume parameter, called CSACVL, is always defined in cubic angstroms and five molecular component sigma profile parameters (SGPRF1−SGPRF5). In this study, these six parameters were all generated using the COSMO*therm* program (S1 Fig in S1 File). In addition to these parameters, the COSMO-SAC model requires a set of pure component physical properties which were determined experimentally and theoretically, using Group contribution methods.

## Results and discussion

### COSMO-RS model

#### COSMO-RS model evaluation

**Fig 6:**
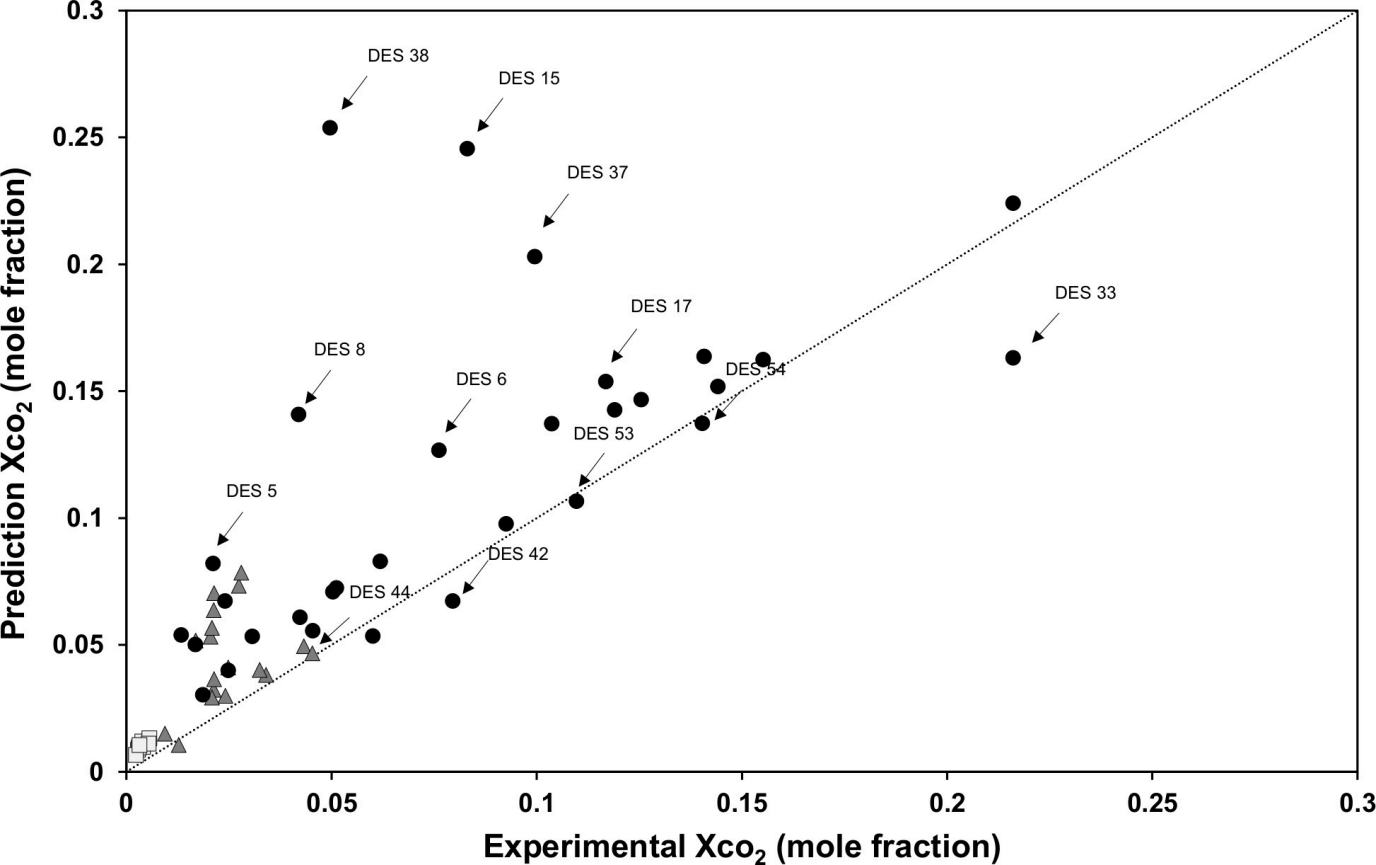
COSMO-RS predictions versus reported experimental values of CO_2_ solubility in DESs. CO_2_ solubility at total pressures of (■) P < 5 bar, (▲) P = 5–9 bar, (**●**) P > 9 bar and (….) identity line *y = x*.

To inspect the accuracy of COSMO-RS predictions of the solubilities of carbon dioxide gas, 58 different types of DESs (S10 Table in S1 File) representing different types of salts, HBDs, and molar structures from the open literature as solvents, and CO_2_ was chosen as the model gas for evaluation. The COSMO-RS model predictions of CO_2_ solubilities in all 58 DES systems presented in S1 Table in S1 File are compared with reported experimental data at different pressures, namely P <5 bar, P = 5–9 bar and P >9 bar, as shown in Fig 6. The average absolute error (AAE) and root mean square deviation (RMSD) were chosen to measure the accuracy of the predictive model, as defined in Eqs 2 and 3, respectively.

$$AAE=\frac{1}{N}\sum_{i=1}^{N}\left(x_{exp.}-x_{cal.}\right)$$ (2)

$$RMSD=\sqrt{\frac{1}{N}\sum_{i=1}^{N}{\left(x_{exp.}-x_{cal.}\right)}^{2}}$$ (3)

**Fig 7:**
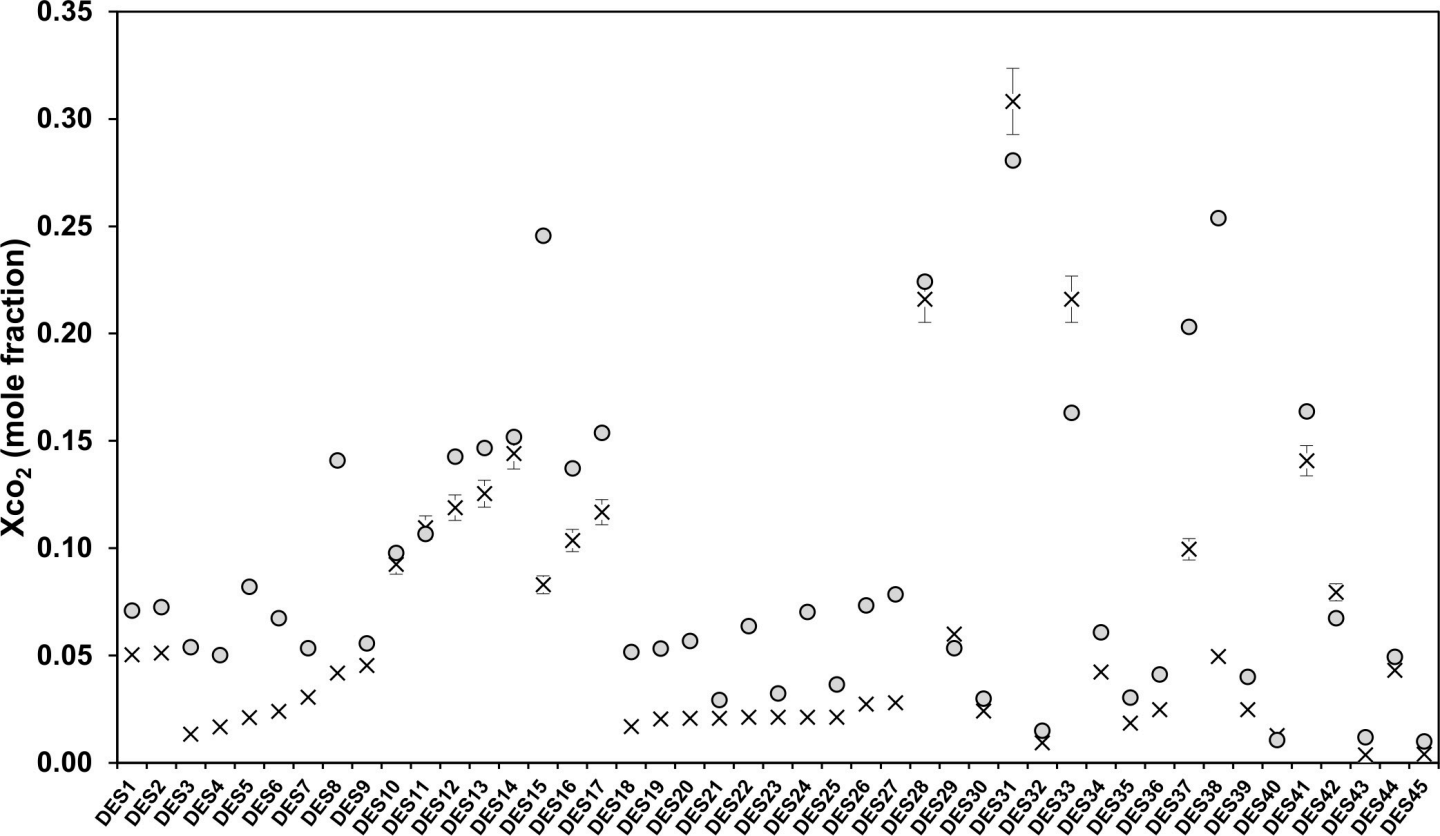
Trends in experimental vs. prediction solubilities of CO_2_. (**x**) Experimental solubility data of CO_2_, (●) COSMO-RS predicted data. Error bars represent the typical 5% error.

The scatter plots in Fig 6 show the systematic error of the distribution between the predicted and experimental CO_2_ solubility data. In fact, the calculated solubilities are systematically over-predicted when compared with experimental data. The predicted and experimental values gave AAEs ranging from 0.005 to 0.028 and RMSDs of 0.01 to 0.1. The AAE of the solubility of CO_2_ at P < 5 bar is 0.005 and the RMSD is 0.019, whereas the predictions of CO_2_ solubility at P > 9 bar achieve the lowest accuracy of 0.15 RMSD and 0.03 AAE. These results confirm that the COSMO-RS model is able to provide good predictions only at low pressure as suggested by Kamgar *et al*. [30]. More specifically, for amine-based DESs (e.g., DES15 and DES17), it was found that the prediction values were mostly more than the values reported in the literature. Furthermore, for ChCl-based DESs (e.g., DES5, DES6, DES8, DES37, and DES38), the prediction were extremely over estimated by the COMSO-RS model. On the other hand, DESs for the choline chloride salt were underestimated (DES33 and DES42) by the model as well. For TBAB- and TBAC-based DESs (DES 44, DES 53, and DES54), it was found that there is a systematic correlation between predicted and literature values. Hence, DESs based on this salt are featured as potential candidates for reliable predicted data in the screening phase. COSMO-RS demonstrates the usefulness of qualitative predictions of the solubilities of CO_2_ as shown in Fig 7. The comparison with experimental trends shows that the model mostly accurately predicts the effects of changing the molar ratio of the cations and anions of the DESs, temperature, and pressure. However, its prediction is opposite that of the experimental values in some cases, for example, urea-based DESs (DES5 and DES6). For TBAB- and TBAC-based DESs, COSMO*therm* predicts the correct order of the HBDs as illustrated in Fig 6: [TEA] < [MEA] < [DEA].

#### COSMO-RS selection of DESs

**Fig 8:**
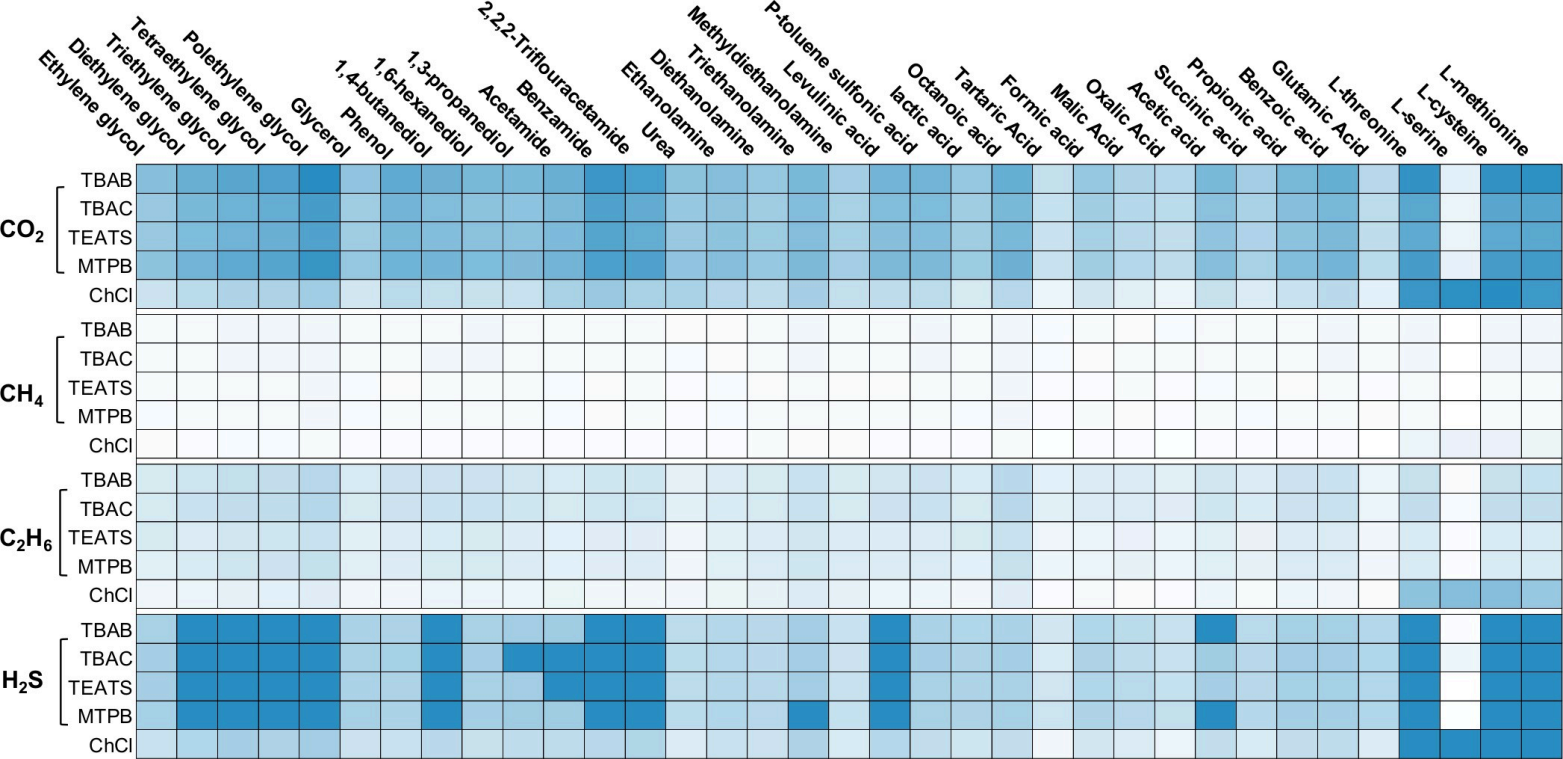
Solubility of CO_2_, CH_4_, C_2_H_6_ and H_2_S in DESs at 25°C and 1 bar for different combinations of salts and HBDs. Solubility index range 0–0.0003 (■), 0.00035–0.1 (■) and 0.15–0.5 (■).

Using the salts and HBDs listed in Tables 3 and 4, the solubilities of CO_2_, H_2_S, CH_4_, and C_2_H_6_ were calculated individually for 170 DES at 25°C and 1 bar. Fig 8 shows the solubilities obtained with each DES for CO_2_, H_2_S, CH_4_, and C_2_H_6_. The predicted CO_2_ solubilities are in the range of those of typical physical solvents. DESs composed of TBAB and MTPB recorded the highest solubility values, whereas ChCl-based DESs achieved the highest value at 0.03 mol_CO2_/mol_solvent_, disregarding the values of the 5^th^ group of HBDs (i.e., amino acids), it can be seen that the alcohol glycol family (e.g., mono-, di-, tri-, tetra-, and poly-) and HBDs with a benzene ring in its structure (e.g., benzamide and benzoic acid) as well as some short-chain fatty acids (e.g., octanoic acid) were those that gave the highest solubilities of all acid gases. The amino acid group has been the most effective of all of the HBD groups covered in this work, achieving high solubility values. However, because of its high viscosity, it would not make a good choice for such applications [40]. A large absorption capacity for CO_2_ or H_2_S alone cannot be used to suggest that a DES has the potential for gas treatment applications because it is also likely that it will have a high capacity for CH_4_ and C_2_H_6_. Thus, the selectivity of the DES requires investigation as well.

As far as Henry’s law constants are concerned, the screening procedure demonstrates the same pattern results as for the solubility. It was found that the DESs with polyglycol-based HBDs (e.g., PEG-4 and PEG-8) and TBAB, TEATS, and MTPB salts have high affinity toward CO_2_. For example, the Henry’s law constants for TBAB-PEG-8 and TEATS-PEG-8 are 21.4 and 24.1 bar, respectively, in which the solubility of CO_2_ is ~2-times higher than in the commonly known DES ChCl-urea. Table 6 presents the Henry’s law constants of CO_2,_ solubility, and selectivity of three systems, (i) CO_2_/CH_4_, (ii) CO_2_/C_2_H_6_, and (iii) CO_2_/C_3_H_8_, the residual activity coefficients at infinite dilutions, and the predicted molecular weights at 298.15 K for the top 40% performing DESs. It can be clearly seen that the solubility of CO_2_ in DES is much higher than in CH_4_. However, the selectivity toward C_2_H_6_ and C_3_H_8_ are high.

**Table 6:** Summary of Henry’s law constant (*H*_CO2_), solubility (*x*_CO2_), selectivity (*S*_a/b_), activity coefficients (*ln γ*^*∞*^) and molecular weight (*MW*_DES_) at 25°C of the top 70 DES system.

Salts	HBD	*MW* (g/mol)	*xco*_*2*_ (mol%)	*Hco*_*2*_ (bar)	*S*_*a/b*_			*ln γ*_*i*_^*∞*^		
					CO_2_/CH_4_	CO_2_/C_2_H_6_	CO_2_/C_3_H_8_	CO_2_	CH_4_	C_2_H_6_
TBAB	Ethylene glycol	114.1	0.035	42.8	37.431	4.771	1.122	-0.3976	1.2313	0.7448
	Diethylene glycol	149.4	0.044	32.5	40.731	4.920	1.110	-0.6656	0.9871	0.5418
	Triethylene glycol	184.6	0.049	28.9	38.991	4.522	1.012	-0.7811	0.8066	0.3926
	Tetraethylene glycol	194.2	0.052	27.8	35.321	4.112	1.072	-0.9501	0.7606	0.3126
	Polyethylene glycol	384.5	0.063	21.4	46.370	4.898	1.021	-1.0636	0.5855	0.2435
	Phenol	139.8	0.047	30.7	33.339	4.339	0.785	-0.7195	1.0010	0.5426
	1,4-butanediol	136.6	0.043	33.4	43.735	5.374	1.224	-0.6396	0.9557	0.5265
	1,6-hexanediol	159.0	0.039	37.6	38.605	4.567	1.034	-0.5247	0.9465	0.5064
	1,3-propanediol	125.3	0.040	37.2	32.393	3.886	0.921	-0.5354	1.0786	0.6232
	Acetamide	111.7	0.044	32.5	38.234	4.679	1.076	-0.6643	1.2484	0.7435
	Benzamide	161.4	0.059	23.5	52.860	6.899	1.535	-0.9759	1.0561	0.5639
	2,2,2Triflouracetamide	154.9	0.054	25.9	62.749	8.057	1.749	-0.8852	1.0398	0.5923
	Ethanolamine	113.3	0.036	40.9	57.870	7.011	1.483	-0.4387	1.3374	0.8496
	Diethanolamine	148.6	0.031	49.3	57.626	9.004	2.140	-0.2586	1.2219	0.7246
	Triethanolamine	183.8	0.039	37.8	45.256	5.777	1.311	-0.5204	0.9238	0.4769
	Levulinic acid	157.4	0.041	35.6	30.436	3.934	0.977	-0.5779	1.0664	0.5868
	P-toluene sulfonic acid	202.2	0.042	34.9	31.000	3.752	0.898	-0.5976	0.9682	0.4862
	Lactic acid	127.6	0.031	49.1	24.511	3.224	0.852	-0.2641	1.2784	0.7584
	Octanoic acid	179.8	0.045	31.9	38.534	4.867	1.134	-0.6841	0.6446	0.2696
	Acetic acid	112.5	0.039	37.7	34.691	4.395	1.061	-0.5208	1.1158	0.6524
	Propionic acid	123.7	0.041	36.1	32.029	4.263	1.060	-0.5617	1.0069	0.5657
	Benzoic acid	162.2	0.045	32.0	29.291	3.229	0.757	-0.6796	0.9311	0.4747
TBAC	Ethylene glycol	105.2	0.030	50.80	28.545	3.542	0.871	-0.2306	1.1692	0.7033
	Diethylene glycol	140.5	0.039	37.85	32.286	3.822	0.895	-0.5189	0.9395	0.5097
	Triethylene glycol	175.7	0.043	33.94	31.390	3.605	0.838	-0.6265	0.7869	0.3804
	Polyethylene glycol	375.6	0.055	25.57	36.599	3.824	0.826	-0.8985	0.5602	0.2266
	Phenol	130.9	0.041	35.60	25.865	3.287	0.836	-0.5771	0.9539	0.5116
	1,4-butanediol	127.7	0.037	40.14	34.913	4.201	0.991	-0.4620	0.9175	0.5015
	1,6-hexanediol	150.1	0.034	44.30	29.663	3.449	0.817	-0.3655	0.9071	0.4805
	1,3-propanediol	116.5	0.034	45.07	25.463	3.002	0.742	-0.3486	1.0402	0.5987
	Acetamide	102.8	0.039	38.05	29.046	3.492	0.843	-0.5116	1.1769	0.6953
	Benzamide	152.5	0.052	27.06	40.712	5.155	1.193	-0.8417	1.0160	0.5378
	2,2,2Triflouracetamide	146.0	0.046	30.97	50.947	6.424	1.440	-0.7134	0.9920	0.5609
	Ethanolamine	104.4	0.033	45.61	44.435	5.269	1.161	-0.3342	1.3037	0.8278
	Diethanolamine	139.7	0.028	54.42	45.337	6.790	1.667	-0.1625	1.2014	0.7117
	Triethanolamine	174.9	0.037	40.74	38.399	4.826	1.125	-0.4468	0.8941	0.4567
	Levulinic acid	148.5	0.037	40.09	26.414	3.380	0.862	-0.4627	1.0050	0.5448
	P-toluene sulfonic acid	193.3	0.038	39.12	27.441	3.281	0.801	-0.4867	0.9249	0.4574
	Lactic acid	127.6	0.028	55.91	21.702	2.809	0.757	-0.1361	1.2068	0.7100
	Octanoic acid	171.0	0.039	37.47	31.410	3.869	0.929	-0.5295	0.6244	0.2570
	Acetic acid	103.6	0.034	43.93	28.924	3.596	0.894	-0.3725	1.0479	0.6063
	Succinic acid	150.1	0.025	62.94	25.468	3.290	0.847	-0.0188	1.3031	0.7645
	Propionic acid	114.8	0.035	42.46	23.568	2.573	0.627	-0.4061	0.9530	0.5296
	Benzoic acid	153.3	0.040	36.75	17.247	2.634	0.804	-0.5469	0.8871	0.4455
TEATS	Ethylene glycol	89.9	0.030	39.99	28.545	3.542	1.019	-0.2306	0.7033	1.1692
	Diethylene glycol	125.1	0.038	32.58	43.644	6.068	0.986	-0.4808	0.7797	1.3332
	Triethylene glycol	160.3	0.042	29.53	40.079	5.205	0.903	-0.6108	0.5842	1.0852
	Polyethylene glycol	360.2	0.051	24.08	44.064	5.224	0.846	-0.8308	0.4370	0.8679
	Phenol	115.5	0.040	30.28	38.814	5.937	0.977	-0.5358	0.8495	1.4483
	1,4-butanediol	112.3	0.036	33.33	51.330	7.569	1.046	-0.4439	0.7534	1.2872
	1,6-hexanediol	134.7	0.034	36.02	40.202	5.449	0.899	-0.3729	0.7033	1.2350
	1,3-propanediol	101.1	0.034	35.88	34.347	4.642	0.821	-0.3516	0.8734	1.4434
	Acetamide	87.5	0.038	32.35	41.681	5.921	0.955	-0.4928	1.0354	1.6712
	Benzamide	137.1	0.050	23.93	61.811	9.565	1.352	-0.8057	0.8571	1.4816
	2,2,2Triflouracetamide	130.6	0.046	26.37	73.642	11.228	1.480	-0.6986	0.8923	1.4763
	Ethanolamine	89.1	0.033	39.91	67.065	9.701	1.239	-0.3281	1.0236	1.5891
	Diethanolamine	124.3	0.029	46.34	78.987	15.424	1.877	-0.1805	0.8555	1.4129
	Triethanolamine	159.6	0.036	37.42	49.140	6.940	1.216	-0.4292	0.5840	1.0804
	Levulinic acid	133.1	0.035	34.54	32.597	4.555	0.928	-0.4008	0.8506	1.4500
	P-toluene sulfonic acid	178.0	0.036	33.29	31.646	4.085	0.846	-0.4399	0.7524	1.3578
	Lactic acid	112.3	0.028	43.45	26.849	3.808	0.824	-0.1361	0.7100	1.2068
	Octanoic acid	155.6	0.039	31.83	43.135	6.366	1.025	-0.5333	0.4338	0.8868
	Acetic acid	88.3	0.033	35.28	39.942	5.941	0.945	-0.3146	0.9579	1.5600
	Propionic acid	99.5	0.034	61.18	25.468	3.290	0.972	-0.3608	0.8422	1.4101
	Benzoic acid	137.9	0.038	24.33	29.808	3.637	0.666	-0.5013	0.7545	1.3406
MTPB	Ethylene glycol	89.9	0.033	51.23	46.570	7.034	1.364	-0.3453	0.9166	1.5822
	Diethylene glycol	125.1	0.042	38.48	43.644	6.708	1.286	-0.6071	0.7275	1.2839
	Triethylene glycol	160.3	0.047	34.53	40.079	5.743	1.112	-0.7385	0.5327	1.0331
	Polyethylene glycol	360.2	0.059	26.88	44.064	6.067	1.077	-0.9847	0.3934	0.8282
	Benzamide	115.5	0.053	38.48	73.642	12.148	2.298	-0.8731	0.8495	1.4732
	Octanoic acid	115.5	0.043	28.98	29.808	3.925	0.798	-0.6294	0.3495	0.8449

#### COSMO-RS experimental validation

Experimental measurements of solubility data for the systems screened in this work are essential. Thus, three DESs with the highest solubilities of CO_2_ were chosen to carry out additional experimental measurements on CO_2_ solubility. The samples were prepared using the protocol described in this work. The experimental values of TBAB+PEG-8 and TBAB+OCT are in good agreement with the data predicted by the COSMO-RS model as shown in Table 7, which confirms that the prediction measurements have good reliability. The predicted values were slightly underestimated by a factor of 1.5 at 1 bar and 1.1 at 2 bar with absolute relative errors (ARD) of 20–40%, which agree with the results obtained by Kamgar *et*. *al*. [30]. Specifically, the experimental value for MTPB+PEG-8 is significantly lower than that predicted by COSMO at 1 bar.

**Table 7:** Comparison between predicted and experimental CO_2_ solubility.

DES	P = 1 bar		P = 2 bar		ARD% ^(b)^
	Exp.^(a)^	Pre.	Exp.^(a)^	Pre.	
TBAB+PEG-8 (1:4)	0.09894	0.06332	0.11176	0.10215	22.3
TBAB+OCT (1:4)	0.08121	0.04517	0.09917	0.07211	35.8
MTPB+PEG-8 (1:10)	0.01250	0.05903	0.08362	0.09177	43.8

^(a)^ Measurements were performed at room temperature.

^(b)^ ARD% = (100/n) ⅀ (*x*exp−*x*_Pre_)/ *x*_exp_ where n is the number of data point.

### ASPEN plus model simulation

The thermodynamic and physical properties determined in this study for potential DES candidates for CO_2_ capture are given in the supporting information; these properties shall be used in the process simulation of the NG sweetening process. The thermophysical properties of the other components (i.e., H_2_S, CO_2_, CH_4_, C_2_H_6_, C_3_H_8_, H_2_O, N_2_, and other HCs) were used from the property databank embedded in ASPEN PLUS without any further investigation. Temperature-dependent properties including density, viscosity, and heat of vaporization of DESs at atmospheric pressure are fitted using the recommended fitting model in ASPEN Plus.

#### ASPEN plus model validation

Before evaluating the performance of the new DES solvent, the developed Aspen model was used to estimate and evaluate the performance of a glycol-based solvent (MEG) and then its results were compared with a baseline case. In this work, the model developed by Alnili and Barifcani [36] was chosen to be the baseline point for comparison. The simulation was conducted using the equilibrium model approach to simulate the absorber and stripper. The number of theoretical stages for the absorber and stripper was set to 40 and 20, respectively, based on the baseline work. The reflux ratio and column duty were selected as column design specifications. The simulation was conducted based on the conditions described above and the parameters presented in Table 5.

**Fig 9:**
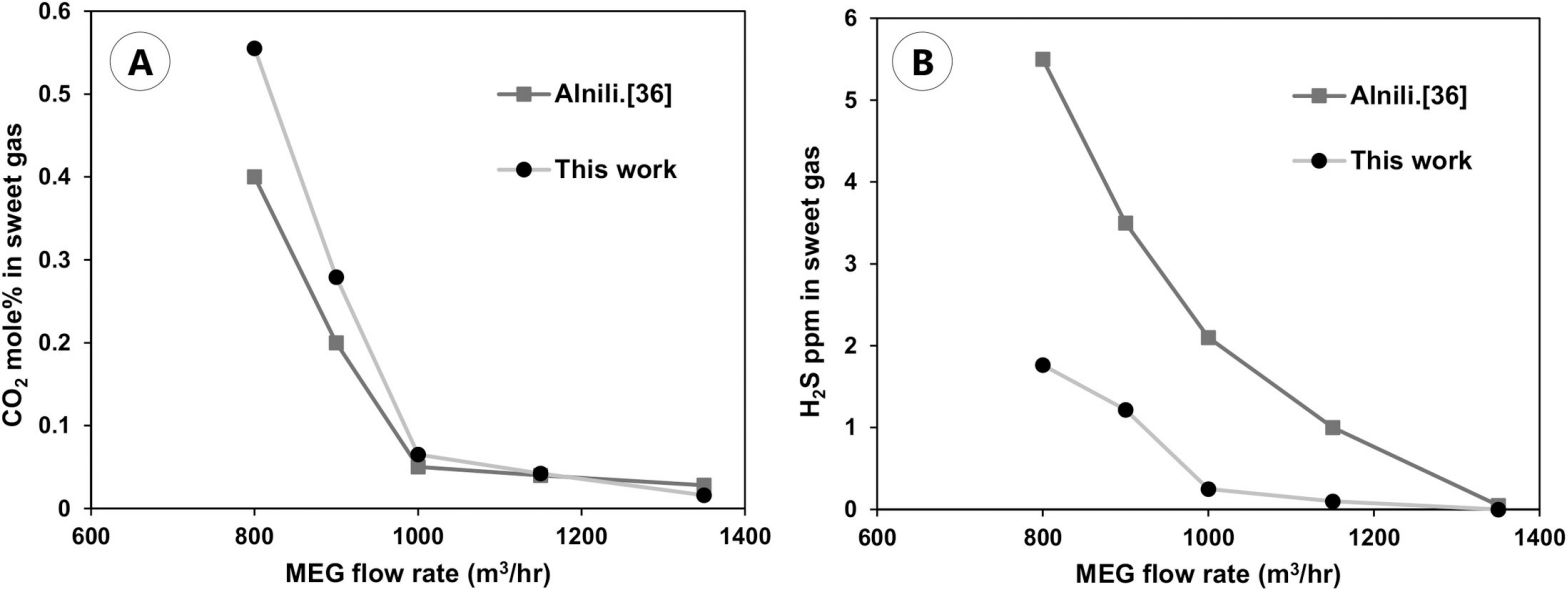
Comparison of the sweetened gas concentration generated in this work with the literature. (A) relationship between MEG flow rate and CO_2_ mol% in sweet gas stream (B) relationship between MEG flow rate and H_2_S ppm in sweet gas stream.

The results presented in Fig 9 show that the gas leaving the absorber (i.e., sweet gas) contains 0.5 mol% CO_2_, 0.04 mol% H_2_O, and 3.3 ppm H_2_S, which matches the results reported in the literature (0.4 mol% CO_2,_ 0.05 mol% H_2_O, and 2.5 ppm H_2_S) [35]. Increasing the MEG circulation rate decreases the amount of acidic gas in the sweetened gas stream as shown in Fig 9. This is intuitive because increasing the solvent circulation rate will cause more gases to be absorbed.

As shown in Fig 9, our results demonstrate similar trends to those reported by Alnili and Barifcani [36], showing excellent agreement for the case of CO_2_ but notable mismatch for H_2_S. The comparison indicated a large deviance in the H_2_S concentration, which can be attributed to the bases of the thermodynamic model chosen in this study and the one used in the literature. The COSMO-SAC model seems to overestimate the solubility of H_2_S in MEG when compared to that in the Peng–Robinson equation of state (PR EoS) model. Hence, we can conclude that the simulation tool in this work is reliable for use in evaluating the performance of new alternative DES solvents, especially for capturing CO_2_.

#### Evaluation of the new alternative solvent

**Fig 10:**
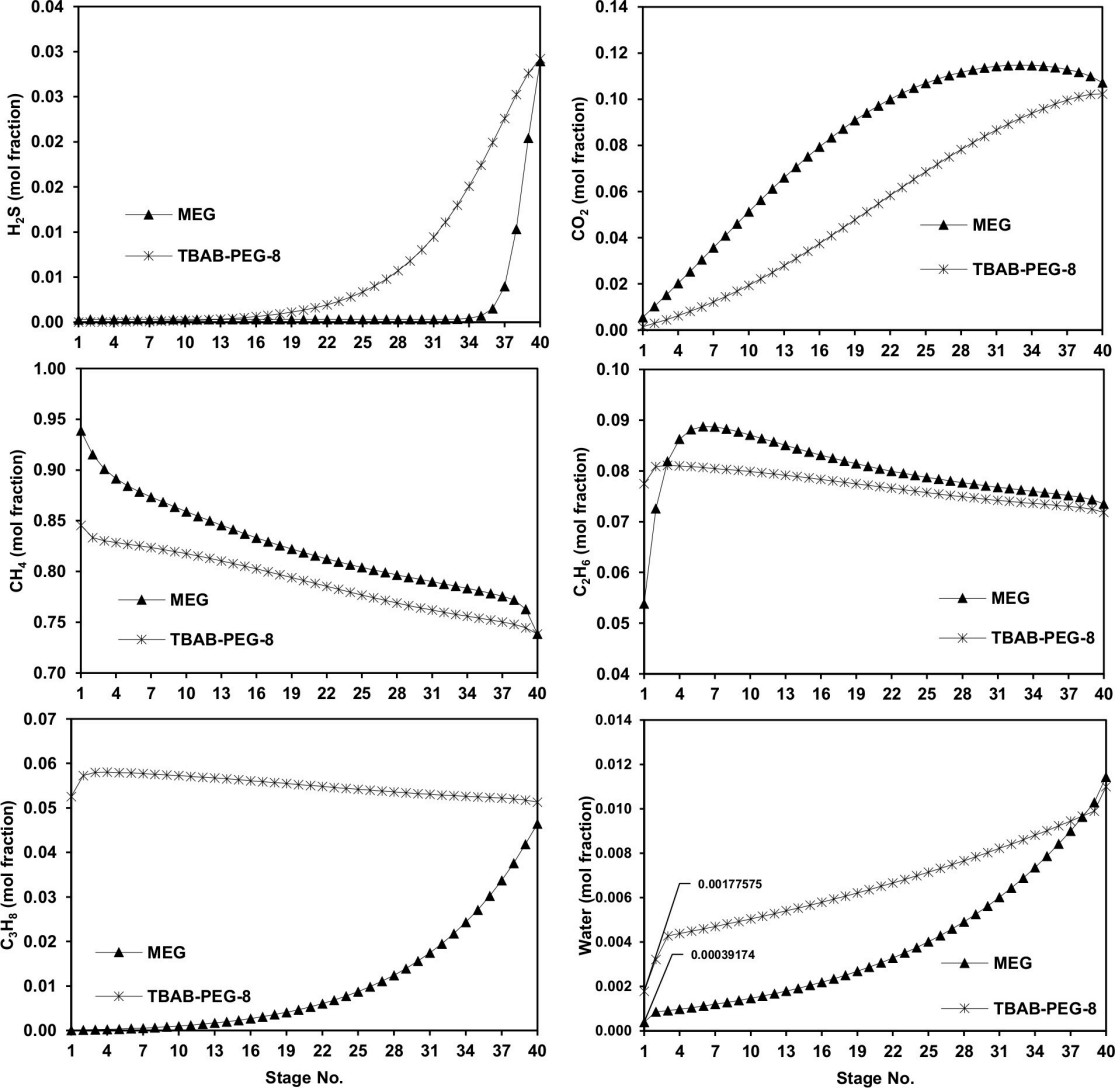
Vapor composition profiles for H_2_S, CO_2_, CH_4_, C_2_H_6_, C_3_H_8_ and water as function of the theoretical number of trays in the absorber.

TBAB+PEG-8 was chosen as a potential alternative to glycol-based solvents for the NG sweetening and dehydration process. The simulation was conducted using the same operating conditions mentioned earlier (Table 6). The H_2_S and N_2_ existing in the raw gas were considered in this study. The solvent should be diluted with water since the measured DES viscosity was relatively high. Hence, dilution will reduce the high pumping power requirements. Simulations were carried out to achieve 1 mol% of CO_2_ and 0.0001 mol% of H_2_S at the absorber outlet gas (sweetened gas) by manipulating the lean solvent flow rate and stripper reflux ratio. The profiles of the vapor mole fraction for different gases (i.e., CO_2,_ H_2_S, CH_4,_ C_2_H_6,_ and C_3_H_8_) inside the absorber are depicted in Fig 10. It is clear that the gas compositions are related to the number of theoretical stages of the absorber. Forty stages is a reasonable value as the concentration of H_2_S in the treated gas (Stage 1) is less than 0.0001 mol% for both TBAB+PEG-8 and MEG at a minimum solvent flow rate of 800 m^3^/h. Additionally, we noted in this figure that the performance of TBAB+PEG-8 is much higher than that of MEG in terms of capturing CO_2_ gas.

**Fig 11:**
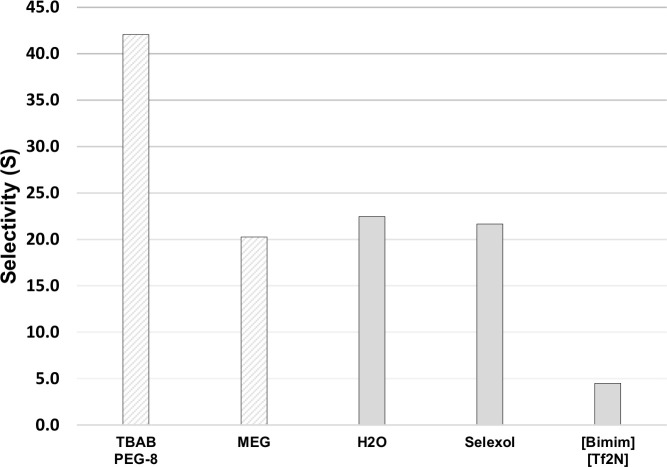
Selectivity of CO_2_ over CH4 for TBAB+PEG-8 and four other physical solvents [41, 42] at 303.2 K and 52 bar.

It was also found that the absorption of TBAB+PEG-8 was able to meet the desired sweet gas concentration specification of 1 mole% at the minimum flow rate. The minimum CO_2_ concentration achieved using TBAB+PEG-8 was 0.15 mol%. The efficiency of CO_2_ separation by DESs was evaluated by the selectivity of CO_2_ over CH_4_, which was calculated from the ratios of mole fractions in the gas phase and in the adsorbed phase. It was found that the selectivity (*S*_CO2/CH4_) of TBAB+PEG-8 outperforms that of MEG and other commercial physical solvents such as H_2_O, Selexol^®^, and other high performance ILs as shown in Fig 11, which makes them attractive candidates for CO_2_ separation in NG sweetening.

Besides the solvent capacity and selectivity performances, the energy consumption involved in the separation process is another essential factor that must be assessed. Assessing energy consumption is vital for such processes because CO_2_ capture by absorption is notorious for being energy demanding. Based on the number of theoretical stages of the absorber (N_th_), the energy assessment was studied based on the ratio of the volumetric flow rate of the absorbent to that of sour gas (L/G) and the assumed flash pressure (P_Flash_) of 5.5 bar. The energy requirements for all units of the gas sweetened process discussed above are listed in Table 8.

**Table 8:** Summary of energy requirements for NG sweetening using proposed DES solvents for the bases of L/G = 0.54, Nth = 40 stage and PFlash = 5.5 bar.

Energy requirements	Units	MEG	TBAB−PEG-8
PUMP (*W*_*P*_)	[kW_e_]	1,620	1,786
FLASH (*Q*_*F*_)	[kW_th_]	4,950	4,662
HEATX-1 (*Q*_*H*_)	[kW_th_]	55,281	44,777
HEATX-2 (*Q*_*C*_)	[kW_th_]	-82,207	-36,748
STRIPPER (*Q*_*H*_)	[kW_th_]	172,117	73,108
Total energy utilization	[kW_e_]	46,600	27,500

The total electrical power was estimated on the basis of the power required for operating the solvent pump (PUMP) with an assumed efficiency of 80% and driver efficiency of 85%. In addition, the heat duty for cooling and heating the NG (HEATX-1 and HEATX-2) and the heat duty of the flash drum (FLASH) as well as the heat duty of the reboiler in the stripper column (STRIPPER) were also considered in estimating the total energy utilization. For the heat duty, a negative value means that cooling duty is required; conversely, heating duty is required. For a better comparison when calculating the total energy utilization, the contributions of the thermal duties (kW_th_) are converted to equivalent electricity duties (kW_e_). For the heating duty, the conversion efficiency of heat to electricity was assumed to be 0.3 [37].

According to the simulation results demonstrated in Table 8, electrical energy requirements for operating the pump are approximately 10–15% higher for DES-based solvents compared to that of MEG, which is ascribed to its high viscosity. The heating requirements were found equally sustained by the cooling requirements of this process. The overall energy performance was found to be reasonably good for the DES-based solvent as the total energy demand decreased by 60% for TBAB+PEG-8 (1:4) when compared to that of MEG. This energy savings is related mainly to the lesser energy demand of the stripper reboiler. The downgraded reboiler duty can be attributed to the fact that physically absorbed species require less energy for regeneration than that required by chemically absorbed species.

#### Comparison of the performance of MEG and DES solvents

The overall performance was examined based on the following criteria: (i) energy performance, (ii) solvent loss, and (iii) CO_2_ removal efficiency. The L/G ratio reflects the solvent circulation rate that is required to meet CO_2_ removal specifications. In this process, a value of 0.54 was fixed for both MEG and TBAB+PEG-8. Thus, a fair comparison can be conducted under the same running environment. The removal efficiency of both solvents was able to achieve the process requirement of 1 mol% of CO_2_ in the sweet gas stream. A summary of the results of all of the process simulations is shown in Table 9. The first observation is that the amount of solvent makeup for the process running with a non-volatile DES-based solvent is equal to zero. The dehydration performance is also considered in this comparison where it was found that MEG has the strongest ability to remove water from the feed gas stream, achieving a sweet gas stream of water content less than ~0.03 mol% (Fig 10) as compared to ~0.17 for TBAB+PEG-8. Thus, TBAB+EG-8 was the most soluble solvent for H_2_S removal as the latter concentration brought the concentration to less than 1.00E-06 mol%, which is 99% below the process target.

**Table 9:** Key parameters result summary.

Parameter	Unit	MEG	TBAB+PEG-8
L/G ratio	[m^3^/ m^3^]	0.54	0.54
Solvent makeup	[kg/h]	110	0.0
CO_2_ recovery	[%]	96.0	99.0
CO_2_ capture degree	[%]	18.0	35.0
Reboiler temperature	[°C]	141.1	90.2
Reboiler duty	[MW_th_]	172	73
Pump duty	[MW_e_]	1.6	1.7
Total energy	[GJ/ton_CO2_]	9.7	5.0
Solvent cost ^(a)^	[$/kg]	0.60	2.78
Sweet gas composition			
H_2_S	[mol%]	<0.0001	<0.00001
CO_2_	[mol%]	0.5	0.1
H_2_O	[mol%]	0.04	0.17

^(a)^ Price quoted form Alfa Aesar Thermo Fisher (Kandel) GmbH.

The energy performance of MEG was found to be the most efficient in terms of power requirements for operating the lean solvent pump. However, it requires much more energy specifically for regenerating the solvent, which increases the total energy requirements of this process to as high as 9.7 GJ/tonCO_2_ compared to 4.9 GJ/tonCO_2_ for TBAB+PEG-8 for the same amount of raw gas supplied.

In addition to its ability to capture 90% of CO_2_ with minimum energy requirements. TBAB+PEG-8 demonstrates high thermal stability in this comparison. Hence, the aqueous TBAB+PEG-8 (1:4) solvent is a promising solvent that could replace MEG in the dehydration and sweetening process.

## Conclusion

In this work, the separation of CO_2_ from sour gas using different DESs was studied through experimental investigation. Moreover, molecular and process simulations were developed in order to screen, design and evaluate process parameters for the system of carbon capture from the sour gas. The molecular simulation, which was conducted with COSMO-RS, was used to evaluate the solubilities of CO_2_ in 170 various DESs at 25°C. From the screening results, new alcohol-based DESs with high solubility and selectivity have been proposed as optional candidates for CO_2_ capture. Moreover, as a result of the screening, three DES (TBAB+PEG-8, MTPB+PEG-8 and TBAB+OCT) were chosen for further experimental measuring their ability to absorb CO_2_ using a VLE apparatus at 25°C and in pressure range from 1 to 2 bar. The experimental values were qualitatively in good agreement with the data predicted by the COSMO-RS model, which proves that the predictions have good reliability. The ARD% between the experiments and predicted solubility were in the range of 20% to 40%.

Finally, the performance of DES for CO_2_ separation was evaluated by conducting process simulation of natural sweetening process using ASPEN PLUS. The simulation results indicated that the DES-based solvent was promising with respect to the amount of solvent loss and the total energy demand for gas sweetening, especially for aqueous TBAB+PEG-8. It was found that the predicted selectivity of these novel solvents for CO_2_ over CH_4_ were higher than the other commercial physical solvent such as Selexol^®^ and MEG as well as other good performance ILs. Therefore, it can be inferred that the aqueous TBAB+PEG-8 (1:4) solvent is a promising candidate that could replace MEG solvent for gas sweetening processes.

## Supporting information

S1 File (DOCX)
