# Supplementary material for: Polyethylene glycol-based deep eutectic solvents as a novel agent for natural gas sweetening
Source: PLoS One. 2020 Sep 21;15(9):e0239493. doi: 10.1371/journal.pone.0239493 (PMC7505472; doi:10.1371/journal.pone.0239493)
Supplement: S1 File — (DOCX) [file pone.0239493.s001.docx]

**Polyethylene glycol-based deep eutectic solvent as a novel agent for natural gas sweetening**

Jiyad N. Aldawsari ^1,2^, Idowu A. Adeyemi ^3^, Abdelbasset Jemai ^1^, Emad Ali ^1^, Inas Al-Nashef ^3^, Mohamed K. Hadj-Kali ^1*^

^1^ Department of Chemical Engineering, King Saud University, Riyadh, Saudi Arabia

^2^ King Abdulaziz City for Science and Technology, Riyadh, Saudi Arabia

^3^ Department of Chemical Engineering, Khalifa University, SAN campus, , Abu Dhabi, United Arab Emirates

* Corresponding Author: [mhadjkali@ksu.edu.sa](mailto:mhadjkali@ksu.edu.sa)

# DES pseudocomponent creation and COSMOSAC property model specification

SGPRF-5

SGPRF-4

SGPRF-1

SGPRF-2

SGPRF-3


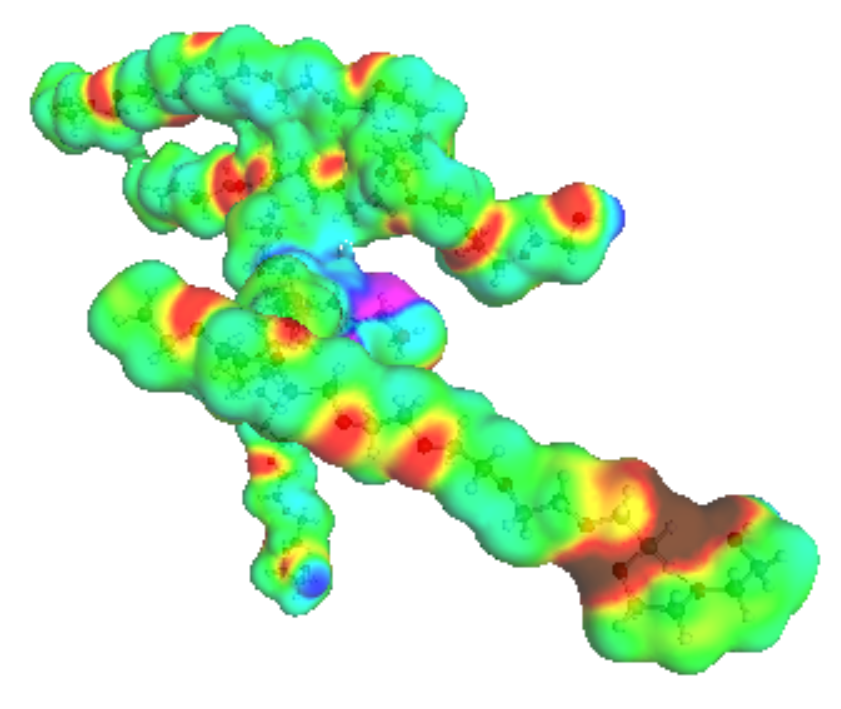


**S1 Figure:** Molecular Shaped Cavity 3D and σ-profile of TBAB+PEG-8 (1:4) obtained using TmoleX v3.5. SGPRF1 to SGPRF5 contains the values of the 12 points delimited in each of the regions across the x-axis

# Implementation of the physicochemical properties of TBAB+PEG-8 (1:4) in Aspen Plus

The correlations needed for the simulation of CO_2_ absorption by glycol-based DES using the COSMO-SAC model are either based on experimental data, empirical correlations or group contribution methods. They are briefly presented and discussed in the following sections:

## Pseudo-critical properties

The critical properties pressure (*P_c_*), temperature (*T_c_*), molar volume (*V_c_*) and compressibility (*Z_c_*) are estimated using the group contribution method proposed by Valderrama *et al*. [1] Their values together with the Aspen Plus parameter code are given in S1 **S1 Table**.

**S1 Table: Critical properties** **of TBAB+PEG-8 (1:4)**

|  | ***M_W_/*g∙mol^-1^** | ***T*_c_ / K** | ***P_c_ /* MPa** | ***V_c_* / cm^3^∙mol^-1^** | ***Z_c_*** |
| --- | --- | --- | --- | --- | --- |
| TBAB+PEG-8 | 360.83 | 1029.02 | 15.90 | 1114.91 | 0.166398 |

## Liquid molar volume

The density of glycol-based DES was determined experimentally as function temperature using the Anton Paar DMA 4100 M vibrating-tube densimeter. The density (*ρ*) was calculated and correlated (S2

**S3 Table**) as function of the temperature using the linear relation given at equation (S2), where *ρ* is in g·cm^3^ and *T* is in K.

| $\rho=a_{1}+b_{1}\cdot T$ | (S1) |
| --- | --- |

**S2 Table: Fitting parameters for the density as a function of temperature**

| **Density** | ***a_1_* / g∙cm^3^** | ***b_1_* / g∙cm^3^∙K^-1^** | ***R^2^*** |
| --- | --- | --- | --- |
| TBAB+PEG-8 | 1.34520 | -0.000782 | 0.9999 |

The liquid molar volume (*v_m_*) was calculated using the density and the molar weight (*v_m_=M_w_/ρ*) and correlated as function of the temperature using the linear relation given at equation (S2), where *v_m_* is in cm^3^·mol^-1^ and *T* is in K.

| $v_{m}=a_{1}+b_{1}\cdot T$ | (S2) |
| --- | --- |

In S3

**S3 Table** the fitting parameters and the coefficient of determination for equation (S2) are presented. The liquid molar volume is implemented in Aspen Plus using the VLPO parameter.

**S3 Table: Fitting parameters for the liquid molar volume VLPO as a function of temperature**

| **VLPO** | ***a_1_* / cm^3^∙mol^-1^** | ***b_1_* / cm^3^∙mol^-1^∙K^-1^** | ***R^2^*** |
| --- | --- | --- | --- |
| TBAB+PEG-8 | 253.38 | 0.2382 | 0.9998 |

## Properties of the normal boiling point

The normal boiling point (*T_b_*) and the acentric factor (*ω*) have been estimated using the group contribution method proposed by Valderrama *et al*. [1] presented in S4 S4 **Table**.

**S4 Table: The properties of the normal boiling point for TBAB+PEG-8 (1:4)**

|  | ***T_b_* / K** | ***Ω*** |
| --- | --- | --- |
| TBAB+PEG-8 | 823.28 | 1.3906 |

## Dynamic viscosity

The dynamic viscosity (*η*) of DES was determined experimentally as function of temperature using the Anton Paar Lovis 2000 ME micro-viscometer. The temperature dependence has been described by the Andrade liquid viscosity equation:

| $\ln\left( \eta\right)=a_{2}+\frac{b_{2}}{T}+c_{2}ln\left( T \right)$ | (S2) |
| --- | --- |

where *η* is given in mPa·s and the temperature in K. S5 **S5 Table** lists the fitting parameters and the coefficient of determination of equation (S2). The Andrade liquid viscosity equation is implemented in Aspen Plus as the MULAND parameter.

**S5 Table: Fitting parameters for the dynamic viscosity (*η*) MULAND as a function of temperature**

|  | ***a_2_*** | ***b_2_* / K** | ***c_2_*** | ***R^2^*** |
| --- | --- | --- | --- | --- |
| TBAB+PEG-8 | -395.1 | 2.19E+04 | 57.38 | 0.9995 |

## Ideal gas heat capacity

The ideal gas heat capacity (*C_p_*^o^) was determined using the Joback group contribution method [2]. The estimated ideal gas heat capacity was represented as a function of temperature by the quadratic equation (S4).

| $C_{p}^{o}=\text{a}_{6}+b_{6}\cdot T+c_{6}\cdot T^{2}$ | (S4) |
| --- | --- |

where the ideal gas heat capacity is given in J·mol^-1^·K^-1^ and the temperature is given in K. The parameters of equation (S4) are used in the CPIGPO Aspen Plus model. The fitted parameters together with their coefficient of determination are given in S6 **S6 Table**.

**S6 Table: Fitting parameters for the ideal gas heat capacity (*C_p_*^o^) CPIGPO as a function of temperature**

|  | ***a_6_* / J·mol^-1^·K^-1^** | ***b_6_* / J·mol^-1^** | ***c_6_* / J·K·mol^-1^** | ***R^2^*** |
| --- | --- | --- | --- | --- |
| TBAB+PEG-8 | 114.99 | 1.3921 | -0.0005 | 0.9999 |

## Molar heat capacity and enthalpy of vaporization

The molar heat capacity of the liquid phase (*C_p_*^l^) has been determined by the group contribution method proposed by Valderrama *et al*. [3]. The parameters of equation (S4) are used in the CPLPO Aspen Plus model. The fitted parameters together with their coefficient of determination are given in S7 **S6 Table**.

**S7 Table: Fitting parameters for the ideal gas heat capacity (*C_p_^L^*) CPLPO as a function of temperature**

|  | ***a_6_* / J·mol^-1^·K^-1^** | ***b_6_* / J·mol^-1^** | ***c_6_* / J·K·mol^-1^** | ***R^2^*** |
| --- | --- | --- | --- | --- |
| TBAB+PEG-8 | 238.59 | 1.9982 | -0.001 | 0.9999 |

The calculated values for the enthalpy of vaporization as function of temperate were correlated to equation (S5), known as the Watson heat of vaporization equation and implemented in Aspen Plus using parameter DHVLWT. The coefficients of the Watson heat of vaporization equation are given in S8 **S8 Table**.

| $\Delta_{vap}\text{H}=\Delta_{vap}\text{H}^{T_{1}}\left(\frac{1-T/T_{c}}{1-T_{1}/T_{c}}\right)^{a_{7}+b_{7}\left(1-\frac{T}{T_{c}}\right)}$ | (S5) |
| --- | --- |

The enthalpy of vaporization and the ideal gas enthalpy of formation were predicted at 298 K. The numerical values of these two properties are presented in **S8 Table**.

**S8 Table: The predicted enthalpy of vaporization (*Δ_vap_H*) and ideal gas heat of formation (*Δ_f_H^ig^*) at 298 K together with the parameters of the DHVLWT equation**

|  | ***T* / K** | ***Δ_vap_H* / kJ·mol^-1^** | ***Δ_vap_H^T1^* / kJ·mol^-1^** | | **DHVLWT** | |
| --- | --- | --- | --- | --- | --- | --- |
|  |  |  |  | *a_7_* | | *b_7_* |
| TBAB+PEG-8 | 298 | 202.11 | -6406.81 | 0.38 | | 0.00 |

## Vapor pressure

The low volatility of the DES was taken into account by setting Aspen Plus extended Antoine vapor pressure equation, PLXANT, to a negligibly small constant value. Equation (S6) is the extended Antoine vapor pressure (*P^vap^* / Pa) used in the simulations. The parameter *a_7_* is given in S9 **S9 Table**.

| $\ln\left(P^{vap}\right)=a_{7}$ | (S6) |
| --- | --- |

**S9 Table: Parameters for the extended Antione vapor pressure equation PLXANT**

|  | ***a_7_*** |
| --- | --- |
| TBAB+PEG-8 | -1.00E-22 |

# Experimental solubility data of DESs

The experimental solubility of CO_2_ for 58 different types of DESs (Fig 6.), representing different type of salts, hydrogen bound donor and molar structure, are listed in S10 Table.

**S10 Table: List of DESs and their experimental CO_2_ solubility**

| **ID** | **Slat** | **HBD** | **Molar ratio** | ***T*/K** | ***P*/MPa** | ***x*_CO2_/mol%** | **Ref.** |
| --- | --- | --- | --- | --- | --- | --- | --- |
| DES1 | Benzyltriphenylphosphonium bromide | Ethylene glycol | 1:12 | 298 | 1.0 | 0.0503 | ^[4]^ |
| DES2 | Benzyltriphenylphosphonium chloride | Glycerol | 1:12 | 298 | 1.0 | 0.0511 | ^[4]^ |
| DES3 | Choline chloride | Ethylene glycol | 1:4 | 298 | 1.0 | 0.0133 | ^[4]^ |
| DES4 | Choline chloride | Ethylene glycol | 1:8 | 298 | 1.0 | 0.0168 | ^[4]^ |
| DES5 | Choline chloride | Urea | 1:2.5 | 298 | 1.0 | 0.0211 | ^[4]^ |
| DES6 | Choline chloride | Urea | 1:4 | 298 | 1.0 | 0.0240 | ^[4]^ |
| DES7 | Choline chloride | Glycerol | 1:8 | 298 | 1.0 | 0.0306 | ^[4]^ |
| DES8 | Choline chloride | Triethylene glycol | 1:4 | 298 | 1.0 | 0.0419 | ^[4]^ |
| DES9 | Choline chloride | Glycerol | 1:3 | 298 | 1.0 | 0.0454 | ^[4]^ |
| DES10 | Choline chloride | Diethanolamine | 1:6 | 298 | 1.0 | 0.0925 | ^[4]^ |
| DES11 | Choline chloride | Ethanolamine | 1:6 | 298 | 1.0 | 0.1096 | ^[4]^ |
| DES12 | Methyltriphenylphosphonium bromide | Ethanolamine | 1:8 | 298 | 1.0 | 0.1189 | ^[4]^ |
| DES13 | Methyltriphenylphosphonium bromide | Ethanolamine | 1:7 | 298 | 1.0 | 0.1254 | ^[4]^ |
| DES14 | Methyltriphenylphosphonium bromide | Ethanolamine | 1:6 | 298 | 1.0 | 0.1441 | ^[4]^ |
| DES15 | Tetrabutylammonium bromide | Triethanolamine | 1:3 | 298 | 1.0 | 0.0830 | ^[4]^ |
| DES16 | Tetrabutylammonium bromide | Diethanolamine | 1:6 | 298 | 1.0 | 0.1036 | ^[4]^ |
| DES17 | Tetrabutylammonium bromide | Ethanolamine | 1:6 | 298 | 1.0 | 0.1168 | ^[4]^ |
| DES18 | Choline chloride | Diethylene glycol | 1:3 | 293 | 0.5 | 0.0168 | ^[5]^ |
| DES19 | Choline chloride | Diethylene glycol | 1:4 | 293 | 0.5 | 0.0205 | ^[5]^ |
| DES20 | Choline chloride | Phenol | 1:2 | 293 | 0.5 | 0.0208 | ^[5]^ |
| DES21 | Choline chloride | Phenol | 1:2 | 323 | 0.5 | 0.0208 | ^[5]^ |
| DES22 | Choline chloride | Phenol | 1:3 | 293 | 0.5 | 0.0212 | ^[5]^ |
| DES23 | Choline chloride | Phenol | 1:3 | 323 | 0.5 | 0.0212 | ^[5]^ |
| DES24 | Choline chloride | Phenol | 1:4 | 293 | 0.5 | 0.0213 | ^[5]^ |
| DES25 | Choline chloride | Phenol | 1:4 | 323 | 0.5 | 0.0213 | ^[5]^ |
| DES26 | Choline chloride | Triethylene glycol | 1:3 | 293 | 0.5 | 0.0274 | ^[5]^ |
| DES27 | Choline chloride | Triethylene glycol | 1:4 | 293 | 0.5 | 0.0280 | ^[5]^ |
| DES28 | Choline chloride | Urea | 1:2 | 323 | 6.5 | 0.2160 | ^[6]^ |
| DES29 | Choline chloride | Urea | 1:2 | 323 | 1.00 | 0.0600 | ^[6]^ |
| DES30 | Choline chloride | Urea | 1:2 | 303 | 0.3 | 0.0241 | ^[7]^ |
| DES31 | Choline chloride | Urea | 1:2 | 303 | 5.7 | 0.3081 | ^[7]^ |
| DES32 | Choline chloride | Ethylene glycol | 1:2 | 303 | 0.2 | 0.0093 | ^[8]^ |
| DES33 | Choline chloride | Ethylene glycol | 1:2 | 343 | 6.3 | 0.2160 | ^[8]^ |
| DES34 | Choline chloride | Ethylene glycol | 1:2 | 303 | 1.00 | 0.0422 | ^[8]^ |
| DES35 | Choline chloride | Ethylene glycol | 1:2 | 343 | 1.1 | 0.0185 | ^[8]^ |
| DES36 | Choline chloride | Lactic acid | 1:2 | 303 | 0.8 | 0.0248 | ^[9]^ |
| DES37 | Choline chloride | Lactic acid | 1:2 | 348 | 9.4 | 0.0995 | ^[9]^ |
| DES38 | Choline chloride | Lactic acid | 1:2 | 303 | 1.7 | 0.0496 | ^[9]^ |
| DES39 | Choline chloride | Lactic acid | 1:2 | 348 | 1.9 | 0.0248 | ^[9]^ |
| DES40 | Choline chloride | Glycerol | 1:2 | 303 | 0.2 | 0.0127 | ^[10]^ |
| DES41 | Choline chloride | Glycerol | 1:2 | 343 | 6.3 | 0.1407 | ^[10]^ |
| DES42 | Choline chloride | Glycerol | 1:2 | 303 | 1.2 | 0.0794 | ^[10]^ |
| DES43 | Tetrabutylammonium bromide | Levulinic acid | 1:3 | 303 | 0.1 | 0.0038 | ^[11]^ |
| DES44 | Tetrabutylammonium bromide | Levulinic acid | 1:3 | 333 | 0.6 | 0.0432 | ^[11]^ |
| DES45 | Tetrabutylammonium chloride | Levulinic acid | 1:3 | 303 | 0.1 | 0.0040 | ^[11]^ |
| DES46 | Tetrabutylammonium chloride | Levulinic acid | 1:3 | 333 | 0.6 | 0.0453 | ^[11]^ |
| DES47 | Tetraethylammonium bromide | Levulinic acid | 1:3 | 303 | 0.1 | 0.0030 | ^[11]^ |
| DES48 | Tetraethylammonium bromide | Levulinic acid | 1:3 | 333 | 0.6 | 0.0324 | ^[11]^ |
| DES49 | Tetraethylammonium chloride | Levulinic acid | 1:3 | 303 | 0.1 | 0.0028 | ^[11]^ |
| DES50 | Tetraethylammonium chloride | Levulinic acid | 1:3 | 333 | 0.6 | 0.0340 | ^[11]^ |
| DES51 | Tetrabutylammonium chloride | Lactic acid | 1:3 | 298 | 0.1 | 0.0054 | ^[12]^ |
| DES52 | Tetrabutylammonium chloride | Lactic acid | 1:2 | 298 | 0.1 | 0.0056 | ^[12]^ |
| DES53 | Tetrabutylammonium chloride | Lactic acid | 1:3 | 318 | 2.0 | 0.1403 | ^[12]^ |
| DES54 | Tetrabutylammonium chloride | Lactic acid | 1:2 | 318 | 2.0 | 0.1551 | ^[12]^ |
| DES55 | Tetraethylammonium chloride | Lactic acid | 1:2 | 298 | 0.1 | 0.0031 | ^[12]^ |
| DES56 | Tetraethylammonium chloride | Lactic acid | 1:2 | 318 | 2.0 | 0.0761 | ^[12]^ |
| DES57 | Tetramethylammonium chloride | Lactic acid | 1:2 | 298 | 0.1 | 0.0023 | ^[12]^ |
| DES58 | Tetramethylammonium chloride | Lactic acid | 1:2 | 318 | 2.0 | 0.0618 | ^[12]^ |

# Summary results of Aspen plus model

A short summary of the main streams of the NG sweetening process is presented in S11 Table.

**S11 Table:** **Stream Table of NG sweetening process using TBAB+PEG-1 (1:4) as solvent.**

| Stream Name | Unit | FEEDGAS | LEANSOL | SWEETGAS | RICHSOL | FLASH-V | SOURGAS | BOTTOM |
| --- | --- | --- | --- | --- | --- | --- | --- | --- |
| From |  |  | PUMP | ABSORBER | ABSORBER | FLASH | STRIPPER | STRIPPER |
| To |  | ABSORBER | ABSORBER |  | VALVE-1 |  |  | HEATX-2 |
| Temperature | C | 30 | 29.8876 | 20.5395 | 22.7105 | 30 | 97.75 | 90.2609 |
| Pressure | bar | 52 | 52 | 52 | 3.5 | 5.5 | 1.05 | 1.05 |
| Molar Vapor Fraction |  | 0.990702 | 0 | 1 | 0.116976 | 1 | 1 | 0 |
| Molar Enthalpy | cal/mol | -26280.4 | -66155 | -19009.6 | -62370.8 | -47182.9 | -54514.3 | -62078.8 |
| Molar Entropy | cal/mol-K | -27.4818 | -104.36 | -26.6883 | -94.0143 | -10.0085 | -10.5504 | -118.88 |
| Molar Density | mol/cc | 0.00208171 | 0.0113597 | 0.00212955 | 0.00111233 | 0.000218212 | 3.40E-05 | 0.00869449 |
| Enthalpy Flow | cal/sec | -3.65E+07 | -2.26E+08 | -1.57E+07 | -2.49E+08 | -2.78E+06 | -5.62E+07 | -1.56E+08 |
| Average MW |  | 22.7046 | 86.3122 | 16.5332 | 78.5115 | 32.8555 | 20.5625 | 110.854 |
| Mole Flows | kmol/hr | 5000 | 12312.5 | 2971.49 | 14360.5 | 212.294 | 3712.52 | 9057.72 |
| H_2_S | kmol/hr | 145 | 7.45E-10 | 1.14E-10 | 145 | 37.767 | 75.3934 | 7.45E-10 |
| CO_2_ | kmol/hr | 500 | 4.03E-14 | 45.5914 | 454.409 | 84.9869 | 75.8844 | 4.03E-14 |
| N_2_ | kmol/hr | 5 | 1.07E-26 | 4.8828 | 0.117204 | 0.17494 | 0.000652298 | 1.07E-26 |
| CH_4_ | kmol/hr | 3600 | 4.85E-17 | 2910.47 | 689.526 | 65.531 | 30.0544 | 4.85E-17 |
| C_2_H_6_ | kmol/hr | 350 | 7.60E-14 | 8.55612 | 341.444 | 9.29821 | 60.9374 | 7.60E-14 |
| C_3_H_8_ | kmol/hr | 250 | 2.05E-11 | 5.00E-12 | 250 | 7.79664 | 98.6325 | 2.05E-11 |
| ISOBU-01 | kmol/hr | 35 | 9.89E-11 | 1.07E-11 | 35 | 1.10617 | 19.911 | 9.89E-11 |
| N-BUT-01 | kmol/hr | 35 | 3.94E-10 | 3.23E-11 | 35 | 1.18047 | 22.3773 | 3.94E-10 |
| N-PEN-01 | kmol/hr | 30 | 2.07E-08 | 6.61E-10 | 30 | 1.01387 | 24.5479 | 2.07E-08 |
| H_2_O | kmol/hr | 50 | 9578.51 | 1.98828 | 9646.02 | 3.43905 | 3304.78 | 6323.73 |
| TBABPEG8 | kmol/hr | 0 | 2733.99 | 3.68E-26 | 2733.99 | 8.13E-19 | 2.77E-213 | 2733.99 |

# References

1. Valderrama, J. O.; Forero, L. A.; Rojas, R. E. Critical Properties and Normal Boiling Temperature of Ionic Liquids. Update and a New Consistency Test. *Ind. Eng. Chem. Res.* 2012, *51* (22), 7838–7844.
2. Poling, B. E.; Prausnitz, J. M.; O’connel, J. P. *The Properties of Gases and Liquids*, 5th ed.; MCGRAW-HILL International Editions: New York, 2001.
3. Valderrama, J. O., Toro, A., & Rojas, R. E, Prediction of the heat capacity of ionic liquids using the mass connectivity index and a group contribution method. The Journal of Chemical Thermodynamics, 2011, 43(7), 1068–1073.
4. Emad Alia, Mohamed K. Hadj-Kalia, Sarwono Mulyono, Inas Alnashefa, Anis Fakeeha, Farouq Mjalli, Adeeb Hayyan, Solubility of CO_2_ in deep eutectic solvents: Experiments and modelling using the Peng–Robinson equation of state, chemical engineering research and design, 2014, 92, 1898–1906.
5. Li, G., Deng, D., Chen, Y., Shan, H., & Ai, N, Solubilities and thermodynamic properties of CO_2_ in choline-chloride based deep eutectic solvents. The Journal of Chemical Thermodynamics, 2014, 75, 58–62.
6. Li, X., Hou, M., Han, B., Wang, X., Zou, L , Solubility of CO_2_ in a choline chloride + urea eutectic mixture. J. Chem. Eng. Data, 2008, 53, 548–550
7. Leron, R. B., Caparanga, A., & Li, M.-H, Carbon dioxide solubility in a deep eutectic solvent based on choline chloride and urea at T=303.15–343.15K and moderate pressures. Journal of the Taiwan Institute of Chemical Engineers, 2013, 44(6), 879–885.
8. Leron, R. B., & Li, M.-H, Solubility of carbon dioxide in a choline chloride–ethylene glycol based deep eutectic solvent. Thermochimica Acta, 2013, 551, 14–19.
9. Francisco, M., van den Bruinhorst, A., Zubeir, L. F., Peters, C. J., & Kroon, M. C ,A new low transition temperature mixture (LTTM) formed by choline chloride+lactic acid: Characterization as solvent for CO_2_ capture. Fluid Phase Equilibria, 2013, 340, 77–84.
10. Leron, R. B., & Li, M.-H, Solubility of carbon dioxide in a eutectic mixture of choline chloride and glycerol at moderate pressures. The Journal of Chemical Thermodynamics, 2013, 57, 131–136.
11. Francisco Pena‐Pereira, Jacek Namieśnik, Ionic Liquids and Deep Eutectic Mixtures: Sustainable Solvents for Extraction Processes, ChemSusChem, 2014, 7.
12. Xu, X., Zhao, X., Sun, L., & Liu, X, Adsorption separation of carbon dioxide, methane and nitrogen on monoethanol amine modified β-zeolite. Journal of Natural Gas Chemistry, 2009, 18(2), 167–172.
